# *Tacr3* in the lateral habenula differentially regulates orofacial allodynia and anxiety-like behaviors in a mouse model of trigeminal neuralgia

**DOI:** 10.1186/s40478-020-00922-9

**Published:** 2020-04-07

**Authors:** Wen-Qiang Cui, Wen-Wen Zhang, Teng Chen, Qian Li, Fei Xu, Qi-Liang Mao-Ying, Wen-Li Mi, Yan-Qing Wang, Yu-Xia Chu

**Affiliations:** 1 Department of Integrative Medicine and Neurobiology, Institutes of Integrative Medicine, School of Basic Medical Sciences, Institutes of Brain Science, Brain Science Collaborative Innovation Center, State Key Laboratory of Medical Neurobiology and MOE Frontiers Center for Brain Science, Fudan University, 130 Dong’an Road, Xuhui District, Shanghai, China; 2 Department of Pain Management, Shandong Provincial Qianfoshan Hospital, the First Hospital Affiliated with Shandong First Medical University, Jinan, Shandong China

**Keywords:** Trigeminal neuralgia, Lateral habenula, Neuropathic pain, Anxiety, *Tacr3*

## Abstract

Trigeminal neuralgia (TN) is debilitating and is usually accompanied by mood disorders. The lateral habenula (LHb) is considered to be involved in the modulation of pain and mood disorders, and the present study aimed to determine if and how the LHb participates in the development of pain and anxiety in TN. To address this issue, a mouse model of partial transection of the infraorbital nerve (pT-ION) was established. pT-ION induced stable and long-lasting primary and secondary orofacial allodynia and anxiety-like behaviors that correlated with the increased excitability of LHb neurons. Adeno-associated virus (AAV)-mediated expression of hM4D(Gi) in glutamatergic neurons of the unilateral LHb followed by clozapine-N-oxide application relieved pT-ION-induced anxiety-like behaviors but not allodynia. Immunofluorescence validated the successful infection of AAV in the LHb, and microarray analysis showed changes in gene expression in the LHb of mice showing allodynia and anxiety-like behaviors after pT-ION. Among these differentially expressed genes was *Tacr3*, the downregulation of which was validated by RT-qPCR. Rescuing the downregulation of *Tacr3* by AAV-mediated *Tacr3* overexpression in the unilateral LHb significantly reversed pT-ION-induced anxiety-like behaviors but not allodynia. Whole-cell patch clamp recording showed that *Tacr3* overexpression suppressed nerve injury-induced hyperexcitation of LHb neurons, and western blotting showed that the pT-ION-induced upregulation of p-CaMKII was reversed by AAV-mediated *Tacr3* overexpression or chemicogenetic inhibition of glutamatergic neurons in the LHb. Moreover, not only anxiety-like behaviors, but also allodynia after pT-ION were significantly alleviated by chemicogenetic inhibition of bilateral LHb neurons or by bilateral *Tacr3* overexpression in the LHb. In conclusion, *Tacr3* in the LHb plays a protective role in treating trigeminal nerve injury-induced allodynia and anxiety-like behaviors by suppressing the hyperexcitability of LHb neurons. These findings provide a rationale for suppressing unilateral or bilateral LHb activity by targeting *Tacr3* in treating the anxiety and pain associated with TN.

## Introduction

Among chronic pain conditions, trigeminal neuralgia (TN) is considered to be one of the most severe [9]. TN is a neuropathic disorder causing excruciating unilateral orofacial pain and mood disorders [27]. It is very common for people with TN to show abnormal anxiety before washing their face or eating food [9], and negative emotions subsequent to chronic pain can further exacerbate the pain sensation [3]. Effective treatments are not available because the underlying mechanism of pain and subsequent anxiety has not been elucidated.

The lateral habenula (LHb) has attracted great attention due to its unique location in linking the systems responsible for negative emotion processing and pain modulation [21]. On the one hand, the LHb has been shown to be involved in detecting and processing nociceptive stimulation in acute and chronic pain [37]. Brain blood flow in the LHb was increased after chronic constriction injury of the sciatic nerve in rats [34], and the expression of c-Fos, the marker of neuron activation, was increased in LHb neurons upon painful stimulation [22]. It is also reported that about two thirds of the LHb neurons show a response to nociceptive stimulation, and among these neurons both excitatory and inhibitory patterns have been recorded depending on the intensity of the stimuli [5]. Pain-excitatory and pain-inhibitory LHb neurons were reported in another work [45], and the LHb has been shown to participate in both analgesic and hyperalgesic circuit loops in acute and chronic pain [37]. On the other hand, the LHb has been shown to be a critical node in the pathophysiology of anxiety [8, 24, 35]. Stereological and 3D morphological analysis showed atrophy and reduced numbers of glial cells in the bilateral LHb of rats with chronic stress-related anxiety [17]. Moreover, the LHb was reported to be activated and to contribute to the induction of anxiety-like behaviors in temporomandibular disorders, which are dysfunctions of the orofacial region [26]. However, whether and how the LHb participates in the pathogenesis of pain and anxiety in TN is unknown.

Here, we established a mouse model of TN by partial transection of the infraorbital nerve (pT-ION). A series of experiments, including behavioral testing, whole-cell patch clamp recording, chemicogenetics, and microarray analysis of differentially expressed genes (DEGs) were performed. The results showed that pT-ION induced long-lasting orofacial allodynia, anxiety-like behaviors, and gene expression adaptations and neuron activation in the LHb ipsilateral to the injured trigeminal nerve. Functional blockade of glutamatergic neurons in the unilateral LHb was enough to alleviate pT-ION-induced anxiety-like behaviors but not allodynia, as was rescuing the pT-ION-induced downregulation of *Tacr3*, a gene enriched in the calcium signaling pathway. The anterior cingulate cortex (ACC), prefrontal cortex (PFC), and hippocampus were reported to be related to the induction of negative emotion [25]. Here we found that the downregulation of *Tacr3* occurred only in the LHb and not in the ACC, PFC, or hippocampus. We also observed the concurrent remission in pT-ION-induced allodynia and anxiety-like behaviors by inhibition of bilateral LHb through chemicogenetic methods or by overexpression of *Tacr3*. The present study shows that pT-ION-induced allodynia and anxiety-like behaviors can be differentially modulated by altering *Tacr3* expression and subsequent LHb function.

## Material and methods

### Animals and models

Adult male C57Bl/6 mice at 7–9 weeks of age were purchased from the Experimental Animal Center of the Chinese Academy of Sciences, Shanghai, China. Before experimental manipulations, the mice were acclimated to the controlled conditions (22° ± 1 °C, a 12:12 light–dark cycle, 8 animals per cage, and food and water available ad libitum) for 1 week. Animal procedures performed in the present study were evaluated and approved by the Animal Care and Use Committee of Fudan University and were conducted strictly in accordance with the guidelines of the International Association for the Study of Pain. All efforts were made to minimize the number and suffering of animals. For each experiment, the animals were randomized to either the control or experimental group. We determined the sample size for each experiment based on our previous work.

#### The partial transection of the infraorbital nerve

Mice were placed in the supine position on a surgical pad under anesthesia with sodium pentobarbital (50 mg/kg, intraperitoneally (i.p.). The oral cavity of the mouse was opened and the pT-ION was performed via an intraoral approach. A 5-mm long incision in the left palatal-buccal mucosa beginning from the first molar was made. The tissue was separated with a pair of forceps, and the left ION was gently dissociated with a tiny glass rod with a hooked tip. The deep branches of the ION were ligated with a catgut (4.0, BD171001, Boda Co., Ltd., Shandong, China) and distally cut off using a pair of surgery scissors, and approximately 2 mm of the nerve fibers were excised to prevent regeneration. The catgut was taken out from the bag immediately before use and immersed in 75% alcohol (80,176,961, Sinopharm Chemical Reagent Co., Ltd., Shanghai, China) to keep it sterile. The incision was closed using tissue glue, and the animals were allowed to recover from the anesthesia after the surgery in a warm chamber. The sham-operated animals underwent unilateral nerve exposure without ligation and transection. The animals were treated with daily intramuscular injection of penicillin potassium (14,005, Keda Co., Ltd., Jiangxi, China) at a dose of 50,000 units/kg (dissolved in sterilized normal saline) for 2 consecutive days after surgery. All of the surgical procedures were performed under aseptic conditions, and no severe infections or post-surgical complications were observed. No treatment was given in the naive group, and animals with emaciation or abnormal movement or mental state were excluded from subsequent experiments. According to our previous work [12], the pT-ION-induced mechanical and cold hypersensitivity is stable for at least 28 days (the longest time period that we observed) and does not spontaneously resolve over time within 28 days after surgery.

### Behavioral tests

Behavioral tests were conducted by experimenters blinded to the group allocation. Pain-related behavior in mice was tested before surgery (day 0) and at 7, 14, and 21 days after surgery, and anxiety-like behaviors were tested 2 h after the pain-related behavior test at 7, 14, and 21 days after surgery. For pain-related behavior, the acetone test was performed 30 min after the von Frey test, and for anxiety-like behaviors the elevated plus maze (EPM) test was conducted 10 min after the open field test (OFT).

#### Von Frey test for mechanical allodynia

We performed a less stressful and more precise method of mechanical allodynia testing. All experiments were carried out in a quiet room under a soft light between 9 a.m. and 6 p.m., and the room temperature was kept at 25 °C throughout the behavioral testings. The ipsilateral hairs in the V2 and V3 measured regions of the mice were removed using a hair clipper (HC1066, Philips, Netherlands). The mice were then habituated to a box (8 cm × 8 cm × 10 cm) made of black wire mesh for 30 min per day for 3 days. A series of von Frey filaments (0.07 g, 0.16 g, 0.4 g, 0.6 g, 1.0 g, 1.4 g, and 2.0 g; Stoelting, USA) were applied to the skin in the V2 or V3 area. Each von Frey filament was applied five times at intervals of a few seconds. A period of up to 6 s was set as the cut-off time to avoid tissue damage. A quick withdrawal of the head after the filament became bent was defined as a response, and the size of the filament at which three positive responses were seen out of five stimulations was defined as the pain threshold.

#### Acetone test for cold allodynia

A 50 μl drop of 90% acetone (diluted in distilled water) was applied to the ipsilateral V3 skin through a 25-gauge needle attached to a 1 mL microsyringe (Hamilton, Reno, NV). Special care was taken to avoid touching the skin and to avoid acetone leakage. The animals were habituated for 3 consecutive days and then baseline values were tested. The total orofacial wiping time was obtained within a cutoff time of 2 min. Cold allodynia was considered present if the wiping time after exposure to acetone was increased compared to basal values or to sham-operated animals. Because of the difficulty in avoiding acetone being sprayed into the eyes, we did not administer acetone to the V2 area, i.e. only the secondary cold allodynia but not the primary cold allodynia was tested.

#### Elevated plus maze test

The EPM test was performed according to a previous study [46]. The maze consisted of four arms (5 cm × 30 cm), including two closed arms having 20-cm high walls and two arms left open (open arms). The maze was elevated 40 cm above the floor. Light intensities in the central area and the opened and closed arms were set to 15 lx, 15 lx, and 5 lx, respectively, and the temperature was controlled at 22° ± 1 °C. Mice were placed in the center of the maze facing an open arm and allowed free access to the four arms for 5 min. The central platform was 6 cm × 6 cm, the two open arms were 30 cm × 6 cm, and the two closed arms were 30 cm × 6 cm × 20 cm. A video tracking system and software (Shanghai Mobile Datum Information Technology Company, Shanghai, China) was used to calculate the percentage of open-arm distance ([open distance]/[total distance] × 100), open-arm entries ([open entries]/[total entries] × 100), and open-arm time ([time in open arms]/[time in total arms] × 100).

#### Open field test

The OFT was administrated as described previously [46]. In brief, the mice were individually placed into the center of an open box apparatus (50 cm × 50 cm × 40 cm) with a black floor. The size of the center zone was set at 25 cm × 25 cm. After freely exploring for 5 min, the time spent in the center square and the total and central distances traveled were recorded and analyzed. Results were expressed as the time spent in the center square, the distance traveled in the center square, and the percentage of the center distance ([center distance]/[total distance] × 100). The experiment was performed under controlled conditions (22° ± 1 °C, dim light (15 lx)).

### Virus injection and chemicogenetic inhibition of LHb neurons

For overexpression of *Tacr3* in the LHb, mice were anesthetized with sodium pentobarbital (50 mg/kg intraperitoneally (i.p.)) and placed into a stereotaxic apparatus. The full-length coding sequence of mouse *Tacr3* (NM_021382) in the AAV2/9 vector (pAAV-CMV-EGFP-2A-*Tacr3*-3FLAG, H12378) and the CaMKII-expressing neuron-targeting virus (pAAV overexpressing vector (AOV)-CaMKII-HM4D(Gi)-EGFP-3FLAG, H14324) were designed and purchased from OBIO Technology Co., Ltd. (Shanghai, China). A total volume of 150 nl virus (pAAV-CMV-EGFP-2A-*Tacr3*-3FLAG; ~ 10^12^ infectious units per ml) was injected into the unilateral or bilateral LHb at bregma (− 1.62 mm), midline (±0.45 mm), and the skull surface (− 2.75 mm). For chemicogenetic inhibition of CaMKII-expressing neurons in the LHb, mice were unilaterally or bilaterally injected in the LHb with 150 nl virus (pAOV-CaMKII-HM4D(Gi)-EGFP-3FLAG; ~ 10^12^ infectious units per ml). Mice were allowed 3 weeks for recovery and transgene expression. Mice were then injected with saline or clozapine-N-oxide (CNO, 2.5 mg/kg, Sigma, i.p.), and behavior was tested 45–55 min post injection. Chemicogenetics uses viral vector–mediated expression of the inhibitory muscarinic M4 receptor–based Gi-coupled DREADD (designer receptor exclusively activated by designer drug) hM4D(Gi) under the control of a certain promoter [43]. Gi signaling activates inward rectifying potassium channels, resulting in hyperpolarization and inhibition of neurons, and DREADD can be activated by the pharmacologically inert molecule CNO [43]. CaMKII acts as the promoter of excitatory neurons. Therefore, the injection of virus (pAOV-CaMKII-HM4D(Gi)-EGFP-3FLAG) into the LHb followed by i.p. injection of CNO at certain time points results in silencing of excitatory neurons (glutamate neurons) in the LHb.

### Immunofluorescence

Immunofluorescence was used to verify viral expression in the unilateral and bilateral LHb. Mice were first anesthetized with sodium pentobarbital (100 mg/kg, intraperitoneally (i.p.)) and perfused transcardially with 0.1 M phosphate-buffered saline (PBS) (pH 7.4) followed by ice-cold 4% paraformaldehyde in 0.1 M PBS. Mouse brains were dissected out and post-fixed at 4 °C overnight. The brains were transferred to a 20% sucrose solution for 24 h followed by a 30% sucrose solution for 48 h at 4 °C. A freezing microtome (Leica 2000, Germany) was used to cut the brain into 30 μm thickness. The slices were then washed three times using 0.3% Triton X-100 and cover-slipped. The cell nuclei were stained by 4′,6-diamidino-2-phenylindole (DAPI) Fluoromount-G® (0100–20, Southern Biotech, Birmingham, AL, USA). Because EGFP is spontaneously fluorescent, the slices did not need to be stained with primary and secondary antibodies. A multiphoton laser point scanning confocal microscopy system (FV1000, Olympus, Tokyo, Japan) was used to obtain the images.

### Western blotting

Radio immunoprecipitation assay lysis buffer was used to homogenize the LHb sample on ice, and then the samples were centrifuged at 12,000 × rpm for 20 min. The Pierce bicinchoninic acid (BCA) kit (Thermo Scientific, Rockford, IL) was used to measure the protein concentration in the supernatant. The samples were boiled with protein loading buffer, and 30 μg total protein was separated by 10% sodium dodecyl sulfate-polyacrylamide gel electrophoresis and then transferred onto polyvinylidene fluoride (PVDF) membranes. The PVDF membranes were blocked with 5% skim milk in TBST (20 mM Tris–HCl, pH 7.5, 150 mM NaCl, and 0.05% Tween-20) for 2 h at room temperature. Membranes were incubated with primary antibodies against p-CaMKII (Thr286, 1:1000 dilution, SC-12886-R, Santa Cruz Biotechnology, Dallas, TX, USA) and GAPDH (1:10,000 dilution, 51,332, Proteintech, Manchester, UK) at 4 °C overnight. The PVDF membranes were washed using TBST and incubated with the HRP-conjugated goat anti-rabbit IgG (H + L) secondary antibody (1:10,000 dilution, SA00001–2, Proteintech) for 1 h at room temperature. An ImageQuant LAS4000 mini image analyzer (GE Healthcare, UK) was used to capture the images, and the Quantity One Analysis Software (Version 4.6.2, Bio-Rad Laboratories, Hercules, USA) was used to quantify the band intensities.

### Microarray analysis and real-time PCR (RT-PCR)

#### Tissue collection

For gene expression profiling (*n* = 15 mice per group) and RT-PCR validation (*n* = 8 adult mice per group), mice were anesthetized by i.p. injection of sodium pentobarbital (50 mg/kg) on day 21 after pT-ION surgery. The LHbs were removed and soaked in RNAlater (R0901, Sigma, USA) and then snap-frozen in liquid nitrogen and kept at − 80 °C. Fifteen Hbs were pooled for tissue sampling, whereas for RT-PCR validation two Hbs were used for the final tissue sample.

#### Microarray analysis

Trizol reagent (Invitrogen) and the mirVana micro RNA (miRNA) Isolation Kit (Ambion, Austin, TX, USA) were used to extract total RNA from the mouse LHbs in both the sham and pT-ION groups according to the manufacturer’s protocol. A NanoDrop 2000 spectrophotometer (Thermo Scientific, USA) was used to determine the RNA yield, and agarose gel electrophoresis stained with ethidium bromide was used to evaluate the RNA integrity. The Agilent Mouse Gene Expression Kit (8*60 K, Design ID:014850) was used in this experiment, and data analysis was performed on the six samples (three samples for each group, with 15 Hbs in each sample). Total RNA was transcribed to double-stranded cDNA, synthesized into cRNA, labeled with Cyanine-3-CTP, and then hybridized onto the microarray. After washing, the Agilent Scanner G2505C (Agilent Technologies) was used to scan the arrays, and the array images were analyzed using the Feature Extraction software (version 10.7.1.1, Agilent Technologies). The raw data were analyzed by Genespring, normalized with the quantile algorithm, and flagged as “Detected”. DEGs were defined as genes with a fold change ≥2.0 and a *p*-value < 0.05.

#### Gene ontology (GO) and Kyoto encyclopedia of genes and genomes (KEGG) pathway analyses

The Database for Annotation, Visualization and Integrated Discovery (DAVID) (https://david.ncifcrf.gov/home.jsp) was used to analyze the GO and KEGG pathways. These data were Log2 transformed and median centered using the Adjust Data function of the R package *gplots*. Hierarchical clustering using the R package *average linkage* was then performed, and DAVID assigned these genes into relevant GO biological pathways and KEGG molecular pathways. Related and significant GO biological pathways were identified by EASE score *p*-values < 0.01, and significant KEGG molecular pathways were identified by EASE score *p*-values < 0.05. Higher enrichment and gene counts indicated more important pathways. Finally, tree visualization was performed using Java Treeview (Stanford University School of Medicine, Stanford, CA, USA).

#### Correlation and coexpression analysis

The Search Tool for the Retrieval of Interacting Genes (STRING) database and Pearson’s correlation coefficient were used in the gene-gene interaction and coexpression analysis. In the present study, the interaction of the DEGs was screened using the STRING 10.5 online tool (http://string-db.org). The DEGS with a combined score ≥ 0.2 were selected, and Cytoscape 3.6.1 was used to construct and visualize the gene-gene interaction network.

#### RT-qPCR validation of regulated genes

We performed RT-qPCR to validate that the *Tacr3* expression was changed only in the LHb and not in other negative emotion-related brain areas including the ACC, PFC, and hippocampus. Total RNA from mouse LHb, ACC, PFC, and hippocampus was isolated using Trizol (Sigma), and the Prime Script RT Kit (Takara) was used for reverse transcription. The Takara SYBR Green reagents were used for RT-qPCR with the Applied Biosystems 7300 plus Detection System according to the manufacturer’s instructions. The sequences of the primers used for the RT-qPCR were as follows: *Tacr3* Forward: TTC ATT CTC ACT GCG ATC TAC CA, Reverse: GCC TGC ACG AAA TCT TTT GTT CA; *Mylk3* Forward: ACC ATG TAC TGA CTA CAG GAG G, Reverse: CCA CTG TTC GCA CAG GTA TGT; *Itpka* Forward: ACT GGC AGA AGA TCC GTA CCA, Reverse: CCG GCA GCT TTG AAA CTC C.

### Brain slice electrophysiology

#### Brain slice preparation

Mice were deeply anesthetized with pentobarbital sodium (50 mg/kg, i.p.) and intracardially perfused with ~ 20 ml ice-cold oxygenated modified artificial cerebrospinal fluid (ACSF) that contained 93 mM N-methyl-d-glucamine (NMDG), 2.5 mM KCl, 1.2 mM NaH_2_PO_4_, 30 mM NaHCO_3_, 20 mM HEPES, 25 mM glucose, 2 mM thiourea, 5 mM Na-ascorbate, 3 mM Na-pyruvate, 0.5 mM CaCl_2_, 10 mM MgSO_4_, and 3 mM glutathione (GSH). The pH of the ACSF was 7.3–7.4, and the osmolarity was 300–305 mOsm·kg^− 1^. Coronal slices (300 μm) that contained the LHb were sectioned at 0.18 mm·s^− 1^ on a vibrating microtome (VT1200s, Leica). The brain slices were initially incubated in NMDG ACSF for 10–15 min at 33 °C, followed by HEPES ACSF that contained 92 mM NaCl, 2.5 mM KCl, 1.2 mM NaH_2_PO_4_, 30 mM NaHCO_3_, 20 mM HEPES, 25 mM glucose, 2 mM thiourea, 5 mM Na-ascorbate, 3 mM Na-pyruvate, 2 mM CaCl_2_, 2 mM MgSO_4_, and 3 mM GSH (pH 7.3–7.4, osmolarity 300–305 mOsm·kg^− 1^) for at least 1 h at 25 °C. The brain slices were transferred to a slice chamber for electrophysiological recording and were continuously perfused with standard ACSF that contained 129 mM NaCl, 2.4 CaCl_2_, 3 mM KCl, 1.3 mM MgSO_4_, 20 mM NaHCO_3_, 1.2 mM KH_2_PO_4_, and 10 mM glucose (pH 7.3–7.4, osmolarity 300–305 mOsm·kg^− 1^) at 3–4 ml·min^− 1^ at 32 °C. The temperature of the ACSF was maintained by an in-line solution heater (TC-344B, Warner Instruments). The recorders were blinded to the group identity during recording and analysis.

#### Whole-cell patch clamp recordings

Neurons in the slices were visualized using a 40× water-immersion objective on an upright microscope (BX51WI, Olympus) equipped with infrared-differential interference contrast and an infrared camera connected to the video monitor. Whole-cell patch clamp recordings were obtained from visually identified LHb cells. Patch pipettes (3–5 MΩ) were pulled from borosilicate glass capillaries (VitalSense Scientific Instruments Co., Ltd) with an outer diameter of 1.5 mm on a four-stage horizontal puller (P1000, Sutter Instruments). The signals were acquired via a Multiclamp 700B amplifier, low-pass filtered at 2.8 kHz, digitized at 10 kHz, and analyzed with Clampfit 10.7 software (Molecular Devices). If the series resistance changed more than 20% during the recording, the experimental recording was immediately terminated.

#### Synaptic transmission

Neurons were held at − 70 mV using the voltage clamp mode for recording spontaneous excitatory postsynaptic currents (sEPSCs). The pipettes were filled with intracellular solution that contained 0.5 mM EGTA, 10 mM HEPES, 125 mM K-Gluconate, 15 mM KCL, 10 mM Na_2_-phosphocreatine, 2 mM Mg-ATP, and 0.5 mM Na-GTP. The osmolarity of the solution was adjusted to 285–290 mOsm·kg^− 1^ and the pH was adjusted to 7.2 with KOH. The baseline was recorded for at least 5 min with standard ACSF. Strychnine (5 μM) and bicuculline (10 μM) were added to the standard ACSF to eliminate inhibitory components, and the slices were incubated in this drug solution for at least 10 min before the experiments. The recording of sEPSCs lasted at least 5 min.

### Statistical analysis

All data are shown as the mean ± SEM. For the pain-related experiments, differences between groups for the von Frey and acetone tests were determined using two-way repeated-measures ANOVA (listed in Supplementary Table S1) followed by Tukey’s multiple comparison test. For the anxiety-like behaviors, differences between groups in the EPM and OFT were determined using one-way ANOVA (listed in Supplementary Table S2) followed by Dunnett’s post hoc multiple comparison test. For the RT-PCR and western blotting results, group differences were determined using one-way ANOVA (listed in Supplementary Table S2) followed by Dunnett’s post hoc multiple comparison test. For the electrophysiological experiments and other comparisons between two groups, differences were determined using unpaired Student’s t-test (listed in Supplementary Table S3). *p* < 0.05 was considered as the threshold of significance in all tests. All statistical analyses were performed using GraphPad Prism 6.0 software (San Diego, CA, USA).

## Results

### pT-ION induced unilateral orofacial allodynia and anxiety-like behaviors in mice

**Fig. 1:**
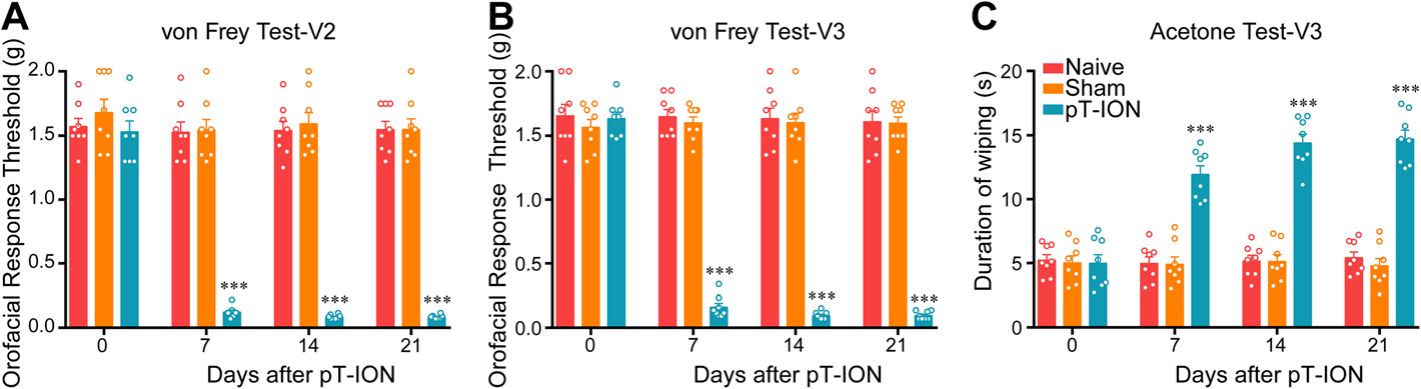
The time course of orofacial allodynia after pT-ION. pT-ION–induced primary allodynia in the ipsilateral V2 skin **(a)** and secondary allodynia in the ipsilateral V3 skin **(b)** as shown by reduced threshold to mechanical stimuli. Secondary cold allodynia was also induced in the V3 skin as shown by increased duration of wiping in response to acetone stimulation **(c)**. *n* = 8 per group, ***p < 0.001 vs. Naive

The trigeminal nerve includes three branches, including the ophthalmic (dominates the orofacial V1 area), the maxillary (the V2 area), and the mandibular (the V3 area) nerves [20]. We previously reported that chronic constriction injury of the infraorbital nerve (CCI-ION) produced long-lasting mechanical allodynia in both the V2 and V3 areas [20]. In this work, we developed the pT-ION model of TN in mice. Consistent with the CCI-ION model, the pT-ION model exhibited both primary allodynia in the injured ION-innervated V2 skin and secondary allodynia in the uninjured V3 nerve-innervated V3 area. The primary and secondary allodynia were constant and persisted for a long time. Mechanical allodynia was measured as reductions compared to baseline in the response threshold to mechanical stimuli applied by von Frey filaments. The ipsilateral orofacial V2 response thresholds of the pT-ION mice were significantly decreased on the 7th day after surgery and lasted for at least 21 days (Fig. 1a), while the V3 secondary nociceptive response decreased over the same time frame (Fig. 1b). In addition to the presence of long-lasting mechanical allodynia, cold allodynia in the V3 area was also elicited using the acetone test. The pT-ION-treated mice had a significantly longer duration of wiping on the orofacial V3 area than that recorded in sham mice at 7 days and lasting for up to 21 days after surgery (Fig. 1c), indicating the occurrence of secondary cold allodynia in the pT-ION model.

**Fig. 2:**
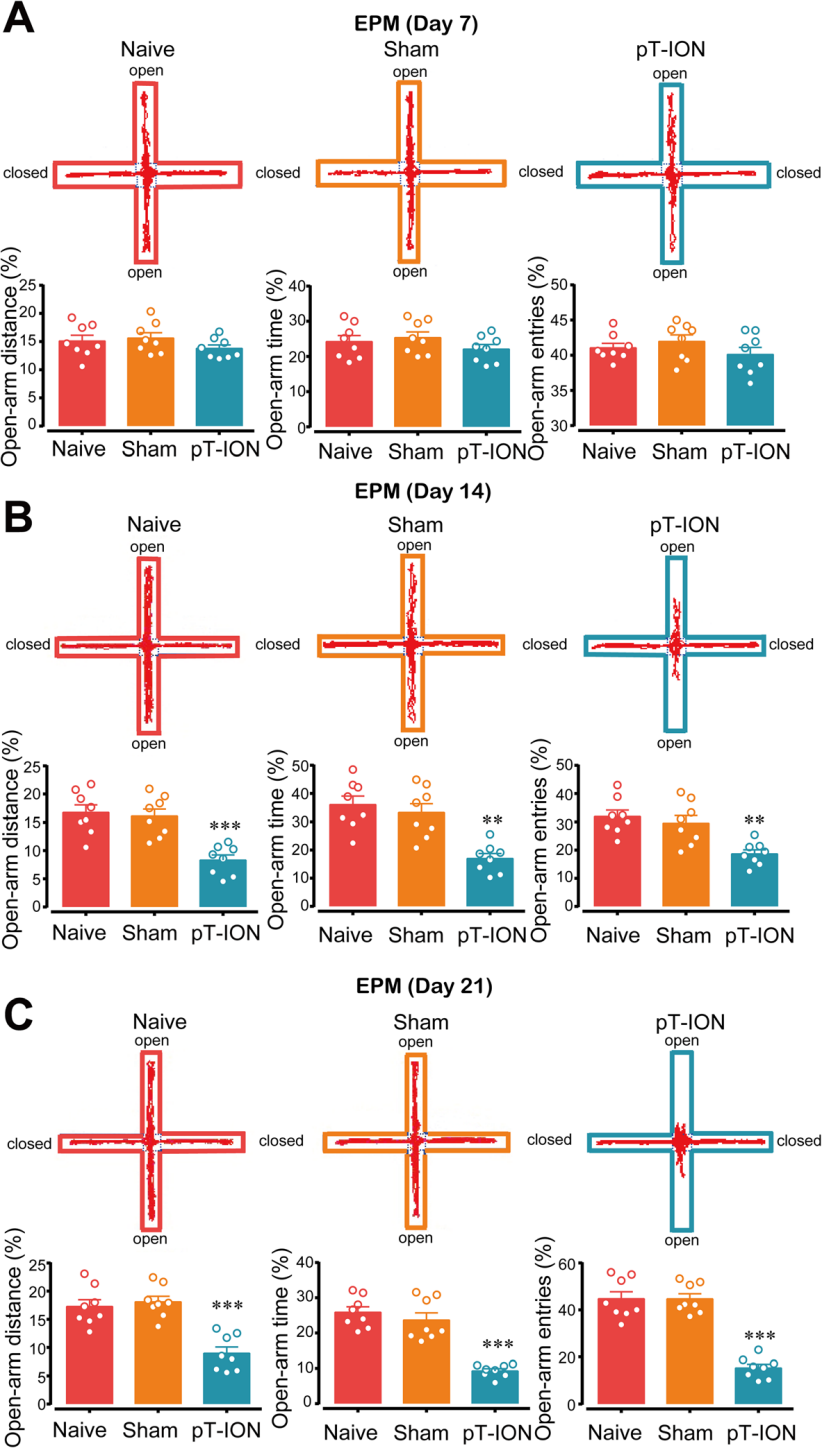
Elevated plus maze test of mice at different time points. The open-arm distance, the open-arm time, and the open-arm entries on day 7 **(a)**, 14 **(b)**, and 21 **(c)** after PT-ION were measured. *n* = 8 per group, **p < 0.01 and ***p < 0.001 vs. Naive

**Fig. 3:**
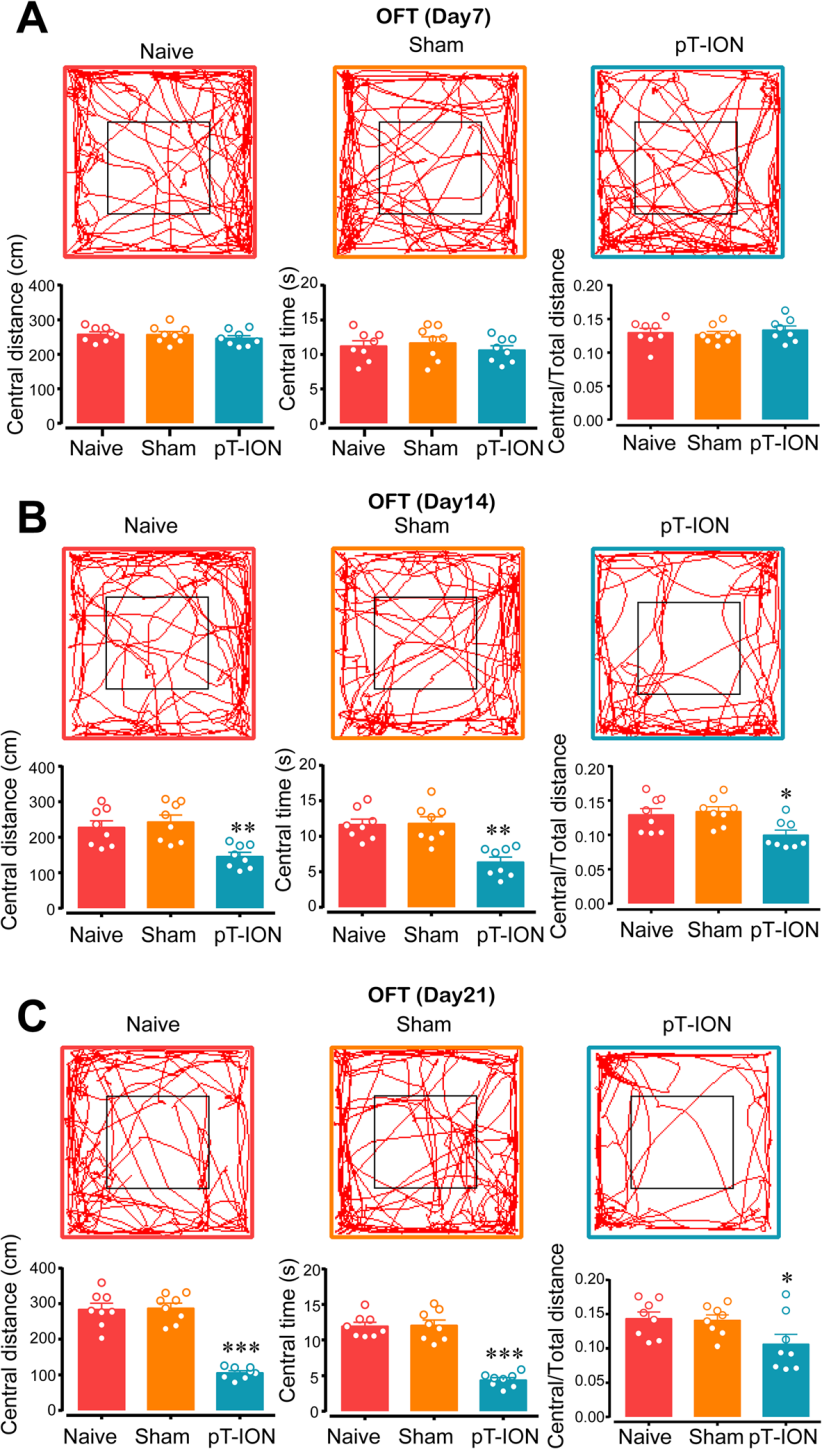
Open field test of mice at different time points. The central distance, the central time, and the central/total distance on day 7 **(a)**, 14 **(b)**, and 21 **(c)** after pT-ION were measured. *n* = 8 per group, *p < 0.05, **p < 0.01, and ***p < 0.001 vs. Naive

To explore whether and when pT-ION-treated mice exhibit anxiety-like behaviors, the EPM and OFT were used at 7, 14, and 21 days after pT-ION. Behavior tests showed that at 14 days and 21 days after nerve injury pT-ION mice had a lower percentage of open-arm distance, time, and entries in the EPM (Fig. 2b and c), but not at 7 days after injury (Fig. 2a). In addition, at 14 days and 21 days after nerve injury the pT-ION mice spent less time in the central area in the OFT and had reduced central distance and central/total distance (Fig. 3b and c), but not at 7 days after injury (Fig. 3a). Together, these results indicated that the trigeminal nerve injury was sufficient to induce anxiety-like behaviors.

### The inhibition of abnormal excitation of unilateral LHb neurons after pT-ION alleviated pT-ION-induced orofacial allodynia but not anxiety-like behaviors

**Fig. 4:**
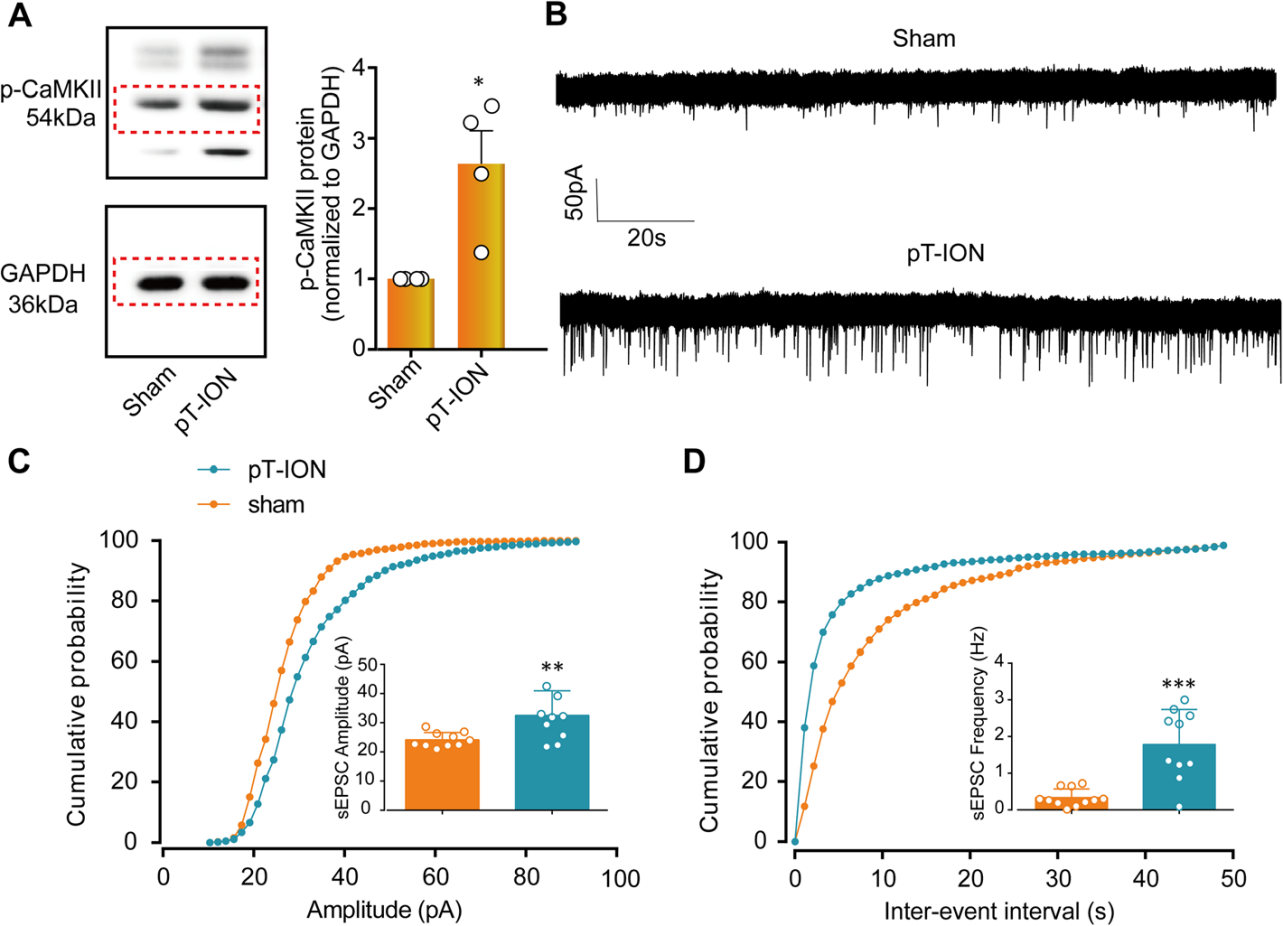
LHb neurons were activated after pT-ION. **(a)** The expression of p-CaMKII in LHb neurons was increased after pT-ION. *p < 0.05 vs. sham. **(b)** Patch-clamp recording showing the typical frequency and amplitude of sEPSCs of sham and PT-ION mice. **(c)** Corresponding cumulative distributions and quantification of sEPSC amplitudes. **(d)** Corresponding cumulative distributions and quantification of sEPSC frequencies. **p < 0.01, ***p < 0.001 vs. sham, *n* = 10 neurons from 4 to 6 mice per group

**Fig. 5:**
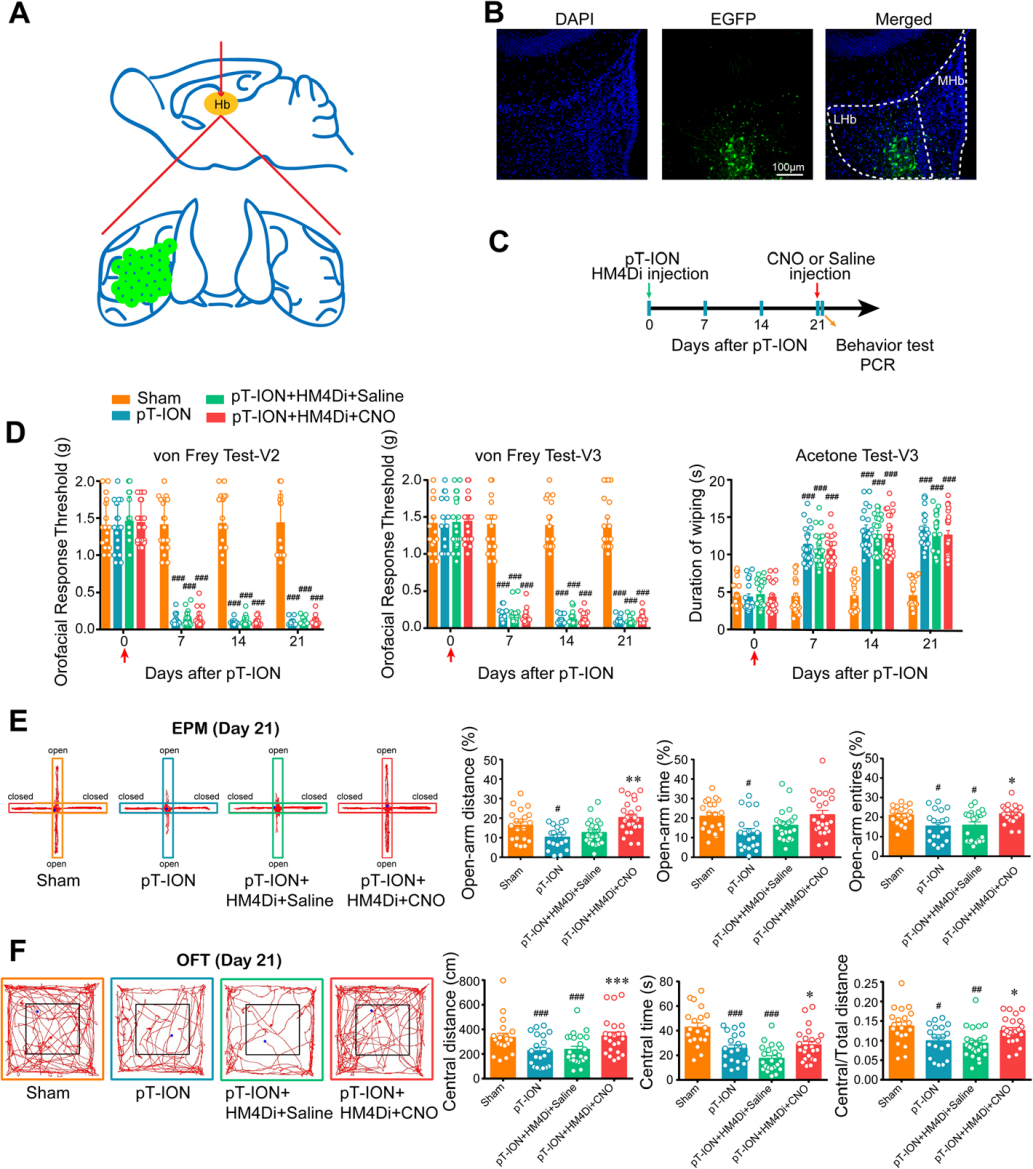
Chemicogenetic inhibition of unilateral LHb neurons alleviated pT-ION-induced anxiety-like behaviors but not allodynia. **(a)** The injection sites of pAOV-CaMKII-HM4Di virus. The green area with black dots shows the virus injection. **(b)** Typical immunofluorescence staining showing the location of virus transfection in the LHb. Green, EGFP; Blue, DAPI. **(c)** The time schedule of pAOV-CaMKII-HM4Di virus and CNO administration, behavioral testing, and PCR. **(d)** Primary mechanical allodynia (left panel), secondary mechanical allodynia (middle panel), and secondary cold allodynia (right panel) were unchanged by pAOV-CaMKII-HM4Di injection in the left LHb and the subsequent CNO application. ### *p* < 0.001 vs. the sham group. **(e)** The percentage of distance and entries but not time spent in the open-arm of the EPM was reversed by the inhibition of left LHb neurons by HM4Di-CNO. # *p* < 0.05 vs. the sham group; *p < 0.05, ** *p* < 0.01 vs. the pT-ION+HM4Di + Saline group. **(f)** The central distance, central time, and central/total distance in the OFT were significantly reversed by the inhibition of left LHb neurons by HM4Di-CNO. # *p* < 0.05, ### *p* < 0.001 vs. the sham group; *p < 0.05, *** *p* < 0.001 vs. the pT-ION+HM4Di + Saline group

To test whether the LHb plays a key role in pT-ION-induced orofacial allodynia and the subsequent anxiety-like behaviors, western blotting and whole-cell patch clamp recording were performed. LHb neurons have been shown to be mostly glutamatergic [2]. The expression of p-CaMKII, a marker of neuronal activation in LHbs, was increased after pT-ION compared with the sham group (Fig. 4a). Whole-cell patch clamp was used to record the sEPSC of the LHb neurons, and the K-S analysis showed that the cumulative probability curve for the amplitude and the inter-event interval shifted to right and left, respectively, after pT-ION, indicating the increase in both the amplitude and frequency of the sEPSC (Fig. 4b-d). Both the western blotting and electrophysiological recording showed that LHb neurons were activated after pT-ION. We then chemicogenetically inhibited the activity of unilateral LHb neurons with HM4Di-CNO. At 21 days after pAOV-CaMKII-HM4Di injection to the left LHb (ipsilateral to the nerve injury), CNO (2.5 mg/kg, i.p.) was administered on its own (Fig. 5a-c). Neither the thresholds for mechanical stimulation in theV2 and V3 skin area nor the duration of wiping after acetone application in the V3 skin area were significantly changed by CNO administration (Fig. 5d). Although no significant difference was detected in time spent in the open-arm in the EPM, the open-arm distance and open-arm entries were significantly increased in the pT-ION+HM4Di + CNO group compared with the pT-ION+HM4Di + Saline group at 21 days after pT-ION (Fig. 5e). In addition, compared with the pT-ION+HM4Di + Saline group, the central distance, central time, and the central/total distance in the OFT were significantly increased in the pT-ION+HM4Di + CNO group at 21 days after pT-ION (Fig. 5f). Both the EPM and OFT suggested that inhibition of the nerve injury-induced hyperexcitation of LHb neurons significantly alleviated the nerve injury-induced anxiety-like behaviors. To determine whether HM4Di + CNO intervention affects the basal pain threshold and emotional behaviors, we injected pAOV-CaMKII-HM4Di into the left LHb of sham mice. Neither the thresholds for mechanical stimulation nor the duration of wiping after acetone application were significantly changed by CNO administration at 21 days after nerve injury (Supplementary Figure S1a), and all parameters for EPM and OFT remained unchanged (Supplementary Figure S1b,c).

### pT-ION-induced DEGs and functional analysis

**Fig. 6:**
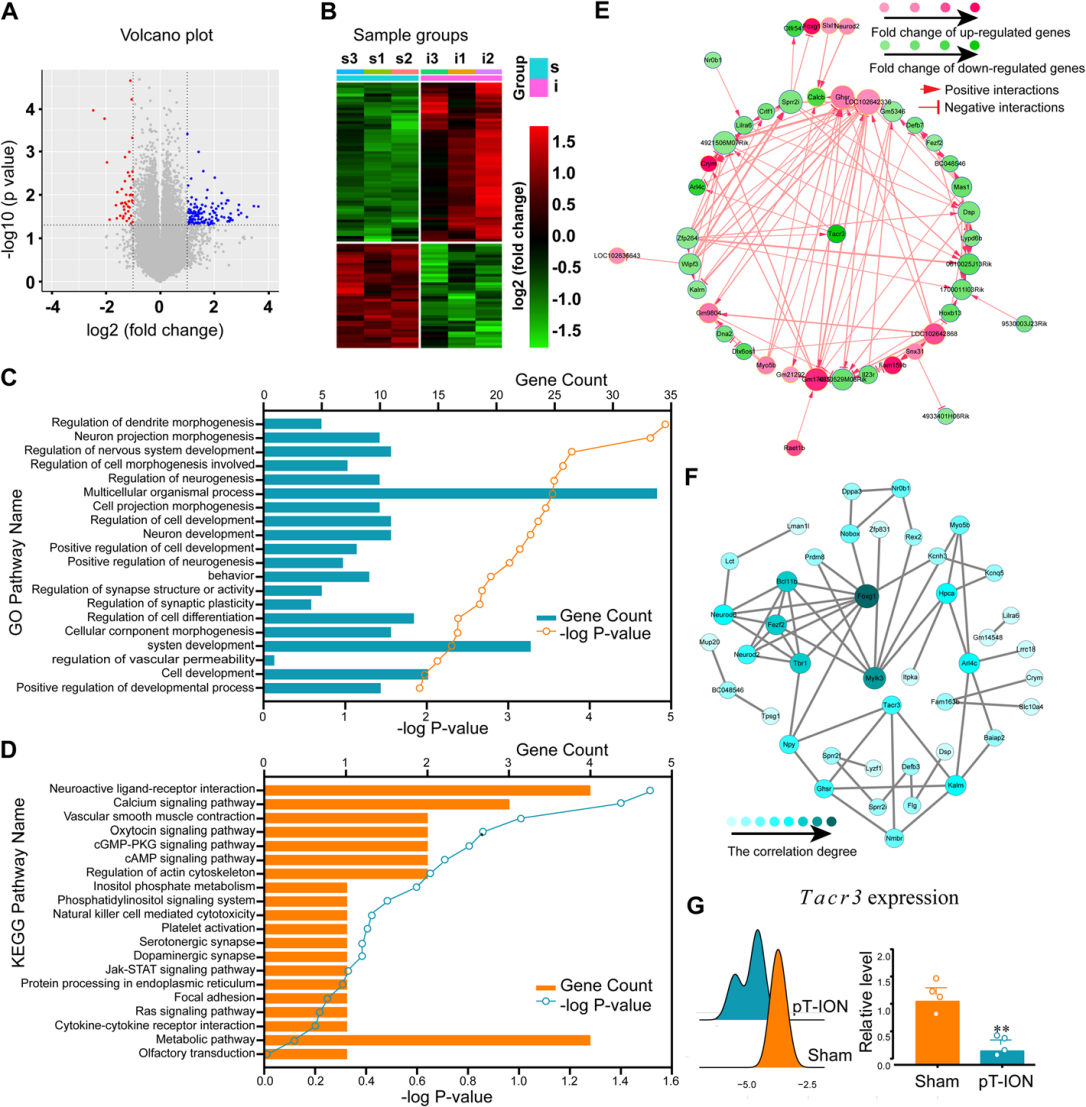
pT-ION-induced gene expression changes in the unilateral LHb and the downregulation of *Tacr3* gene expression. **(a)** The volcano plot is based on the expression values of all genes detected by microarray. **(b)** The heat maps showing DEGs with fold change ≥2.0 (*p* < 0.05). s, sham group; i, nerve injury group. **(c)** GO annotations of DEGs in 20 selected biological processes. **(d)** KEGG pathway enrichment analysis of DEGs in the selected 20 pathways. **(e)** Gene co-expression network. Each circle corresponds to a protein-coding gene (mRNA). The red circles represent up-regulated genes, and the green circles represent down-regulated genes. The size of the circle represents the degree of correlation with other genes, and the stronger the correlation degree, the larger the circle. The color of the circle is determined by the log (fold change) of each gene. Lines ending with arrows and vertical lines represent positive and negative interactions between genes, respectively. **(f)** PPI String network. Circles and lines represent target genes and interactions between genes, respectively. The sizes of the circles represent the degree of correlation degree with other genes, and the stronger the degree of correlation, the larger the circle. The color of the circle is determined by the degree of correlation. **(g)** Ridge map and RT-qPCR validation of pT-ION-induced differential expression of the *Tacr3* gene in the LHb. *n* = 4 samples (8 mice) per group. ** *p* < 0.01 vs. the sham group

Volcano plots were made to assess gene expression changes between the sham and pT-ION groups. In total, 171 DEGs displayed differential expression in the LHb of pT-ION mice, including 126 upregulated DEGs and 45 downregulated DEGs (Fig. 6a). Hierarchical clustering analysis demonstrated overall alterations in the expression of DEGs among the samples (Fig. 6b). The data suggested that the gene expression in the pT-ION group differed from that in the sham controls.

The GO and KEGG pathway analysis of the DEGs might provide clues about the comorbidity of pain-anxiety processes. We utilized all DEGs for the GO analysis and selected 20 functions that we were interested in (Fig. 6c). We found that the significantly enriched GOs included regulation of dendrite morphogenesis, neuron projection morphogenesis, and regulation of nervous system development. In the KEGG pathway analysis, DEGs were found to be mostly enriched in neuroactive ligand-receptor interactions and calcium signaling pathways among the 20 selected pathways that were thought to be associated with the development of pain and mood disorders (Fig. 6d). It should be noted that the calcium signaling pathway was the third most highly enriched pathway.

### The interaction and co-expression network of DEGs in pT-ION mice and the downregulation of *Tacr3* in the calcium signaling pathway

A total of 106 co-expression pairs involving 43 DEGs were filtered (Fig. 6e), and *Tacr3* was among the top 24 genes of the co-expression network in terms of degree of connectivity. In addition, the established protein-protein interaction (PPI) network contained 42 nodes (DEGs) and 60 edges (Fig. 6f). By comparing the two lists of DEGs in the PPI network and co-expression network analyses, we found that one gene in the calcium signaling pathway, *Tacr3*, appeared in both networks. Moreover, *Tacr3* was among the top 20 most strongly up- or down-regulated genes in the pT-ION mice (Supplementary Table S4). All of these results suggested that *Tacr3* might play an important role in terms of TN and its associated mood disorders. This gene belongs to the gene family that encode receptors for tachykinins, which interact with G proteins and have seven hydrophobic transmembrane regions [28]. *Tacr3* encodes neurokinin 3 receptor (NK3R), which has been reported to be related to the modulation of pain hypersensitivity and negative emotion [18, 33]. Thus, the *Tacr3* gene was selected as the focus of our study in the following experiments. Using the *ggridges* function in the R package *ggplot2*, we plotted the ridge map of the *Tacr3* gene. The expression of *Tacr3* in pT-ION mice shifted to the left compared with the expression in sham mice (Fig. 6g, left), indicating the downregulation of *Tacr3* after nerve injury. The pT-ION-induced downregulation was validated by RT-PCR (Fig. 6g, right). In addition, to test whether the down-regulation of *Tacr3* expression was seen only in the LHb, we performed RT-PCR to detect the expression of *Tacr3* in other brain regions. The expression of *Tacr3* was not significantly changed in the ACC (Supplementary Figure S2a), PFC (Supplementary Figure S2b), or hippocampus (Supplementary Figure S2c) after pT-ION, which indicated that the down-regulation of *Tacr3* was specific to the LHb.

### Reversing the downregulation of *Tacr3* in the unilateral LHb alleviated pT-ION-induced anxiety-like behaviors but not orofacial allodynia

**Fig. 7:**
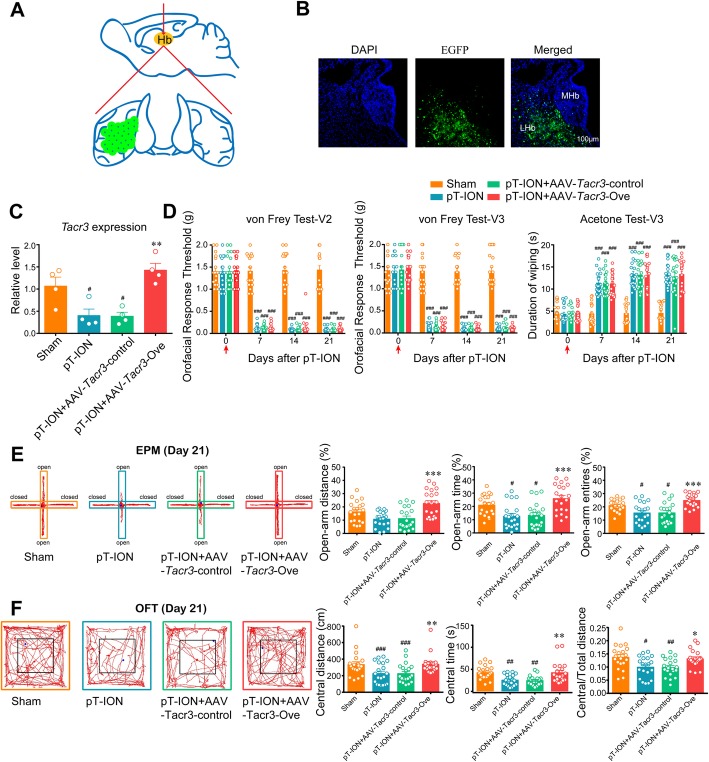
*Tacr3* overexpression in the unilateral LHb improved the pT-ION-induced anxiety-like behaviors, but not the allodynia. **(a)** The virus injection sites in the LHb. The green area and black dots represent the virus injection sites. **(b)** Typical immunofluorescence staining for AAV-*Tacr3*-Ove virus (EGFP, green) and DAPI (blue). **(c)** The expression of *Tacr3* was significantly increased in LHb neurons. # *p* < 0.05 vs. the sham group; ** *p* < 0.01 vs. the pT-ION+AAV-*Tacr3*-control group. **(d)** The primary mechanical allodynia (left panel), secondary mechanical allodynia (middle panel), and secondary cold allodynia (right panel) were not changed by *Tacr3* overexpression. ### *p* < 0.001 vs. the sham group. **(e)** The decrease in percent of the open-arm distance, time, and entries in the EPM were completely reversed by *Tacr3* overexpression. # *p* < 0.05 vs. the sham group; *** *p* < 0.001 vs. the pT-ION+AAV-*Tacr3*-control group. **(f)** The central distance, central time, and central/total distance were significantly increased after *Tacr3* overexpression. # *p* < 0.05, ## *p* < 0.01, ### *p* < 0.001 vs. the sham group; *p < 0.05, ** *p* < 0.01 vs. the pT-ION+AAV-*Tacr3* control group

To test whether the downregulation of *Tacr3* in the unilateral LHb was involved in pT-ION-induced allodynia and anxiety-like behaviors, we overexpressed *Tacr3* by injecting AAV-*Tacr3*-Ove virus into the left LHb (Fig. 7a and b). Compared with the pT-ION+AAV-*Tacr3*-control group, the expression of *Tacr3* was significantly upregulated in the pT-ION+AAV-*Tacr3*-Ove group, indicating that the decrease in *Tacr3* expression was successfully reversed (Fig. 7c). Neither the primary and secondary mechanical allodynia nor the secondary cold allodynia were alleviated by the rescue of *Tacr3* downregulation through AAV-*Tacr3*-Ove virus injection (Fig. 7d). Consistent with previous results (Fig. 5d), unilateral intervention in the LHb was not analgesic. In contrast, in the EPM the open-arm distance, open-arm time, and open-arm entries were significantly increased in the pT-ION+AAV-*Tacr3*-Ove group compared with the pT-ION+AAV-*Tacr3*-control group at 21 days after pT-ION (Fig. 7e). In addition, compared with the pT-ION+AAV-*Tacr3*-control group, the central distance, central time, and central/total distance in the OFT were significantly increased in the pT-ION+AAV-*Tacr3*-Ove group at 21 days after surgery (Fig. 7f). Both the EPM and OFT suggested that the overexpression of *Tacr3* in unilateral LHb neurons was enough to rescue nerve injury-induced anxiety-like behaviors. As we expected, neither the thresholds for mechanical stimulation nor the duration of wiping after acetone application were significantly changed after unilateral overexpression of *Tacr3* in sham mice (Supplementary Figure S3a), and the parameters for the EPM and OFT were unchanged (Supplementary Figure S3b,c). These behavioral tests indicated that the overexpression of *Tacr3* in unilateral LHb did not affect the basal pain thresholds or emotional behaviors of the mice.

### Reversing the downregulation of *Tacr3* suppressed the hyperexcitability of LHb neurons

**Fig. 8:**
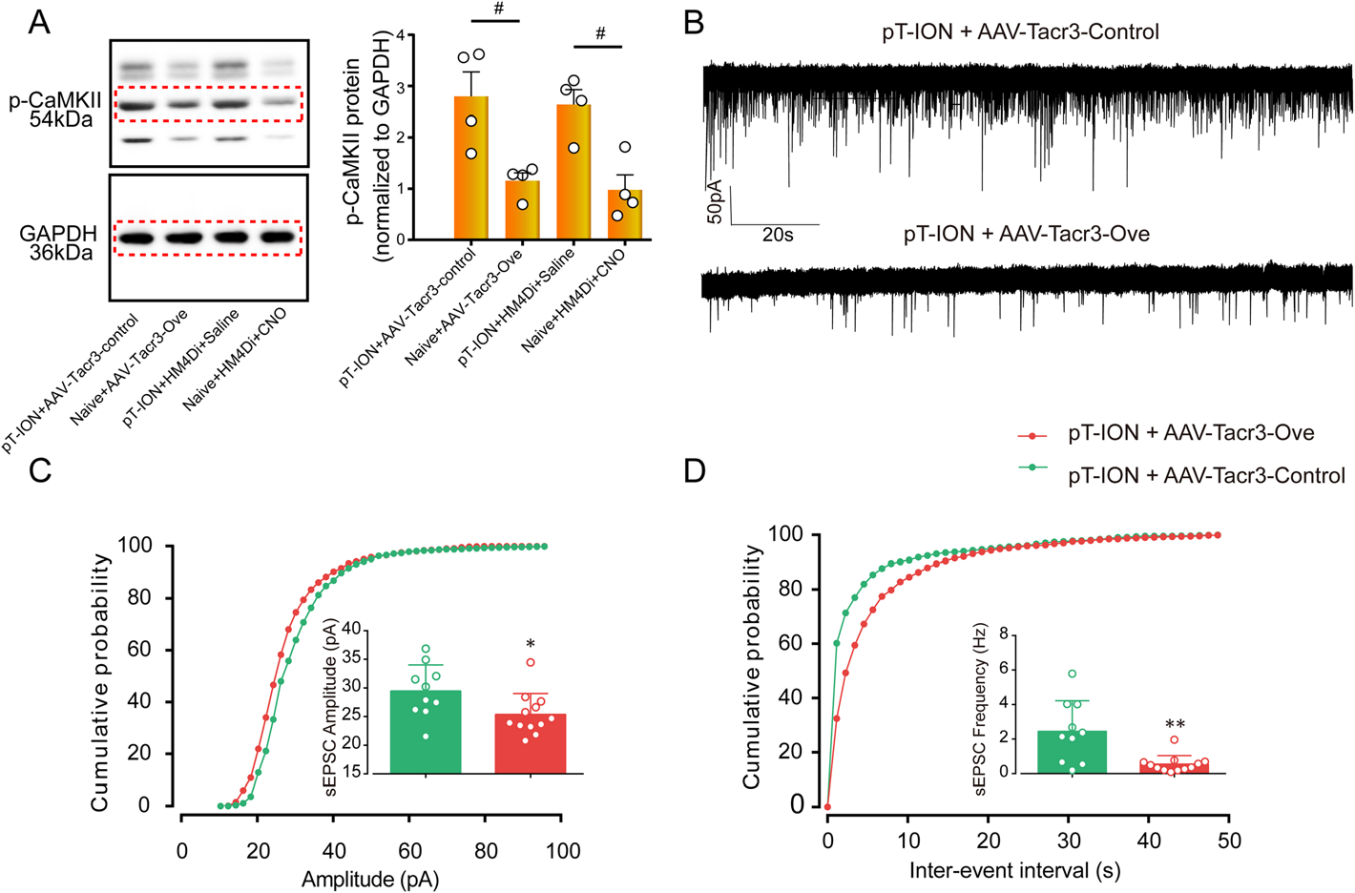
The electrophysiological activity of LHb neurons was suppressed by *Tacr3* overexpression. **(a)** The expression of p-CaMKII in the LHb was decreased after AAV-*Tacr3*-Ove injection and HM4Di-CNO administration. # *p* < 0.05, vs. control. **(b)** Typical current recorded by whole-cell patch clamp showing the suppression of both frequency and amplitude of sEPSCs by *Tacr3* overexpression. **(c)** Corresponding cumulative distribution and quantification of sEPSC amplitudes. **(d)** Corresponding cumulative distribution and quantification of sEPSC frequencies. *p < 0.05, **p < 0.01 vs. the pT-ION+AAV-Tacr3-control group, *n* = 10 neurons from 4 to 6 mice per group

Compared with the pT-ION+HM4Di + Saline group, the expression of p-CaMKII was significantly decreased after HM4Di-CNO treatment (Fig. 8a). We showed that pT-ION-induced hyperexcitability of LHb neurons (Fig. 4) and chemicogenetic inhibition of LHb neuronal activity alleviated the pT-ION-induced anxiety-like behaviors (Fig. 5). It was hypothesized that *Tacr3* dysregulation plays a role in regulating LHb neuron excitability. To test this hypothesis, we performed whole-cell patch clamp recording of the sEPSC in LHb neurons. Compared with the pT-ION+Tacr3-control group, both the amplitude and frequency of sEPSC of LHb neurons in the pT-ION+Tacr3-Ove group were significantly decreased (Fig. 8b-d), indicating the inhibition of LHb neuronal hyperexcitability by *Tacr3* overexpression. Western blotting also showed that the expression of p-CaMKII was significantly decreased after HM4Di-CNO treatment and *Tacr3* overexpression compared with the control group (Fig. 8a).

### Inhibition of bilateral LHb neurons by chemicogenetic methods or *Tacr3* overexpression reversed both allodynia and anxiety-like behaviors induced by pT-ION

**Fig. 9:**
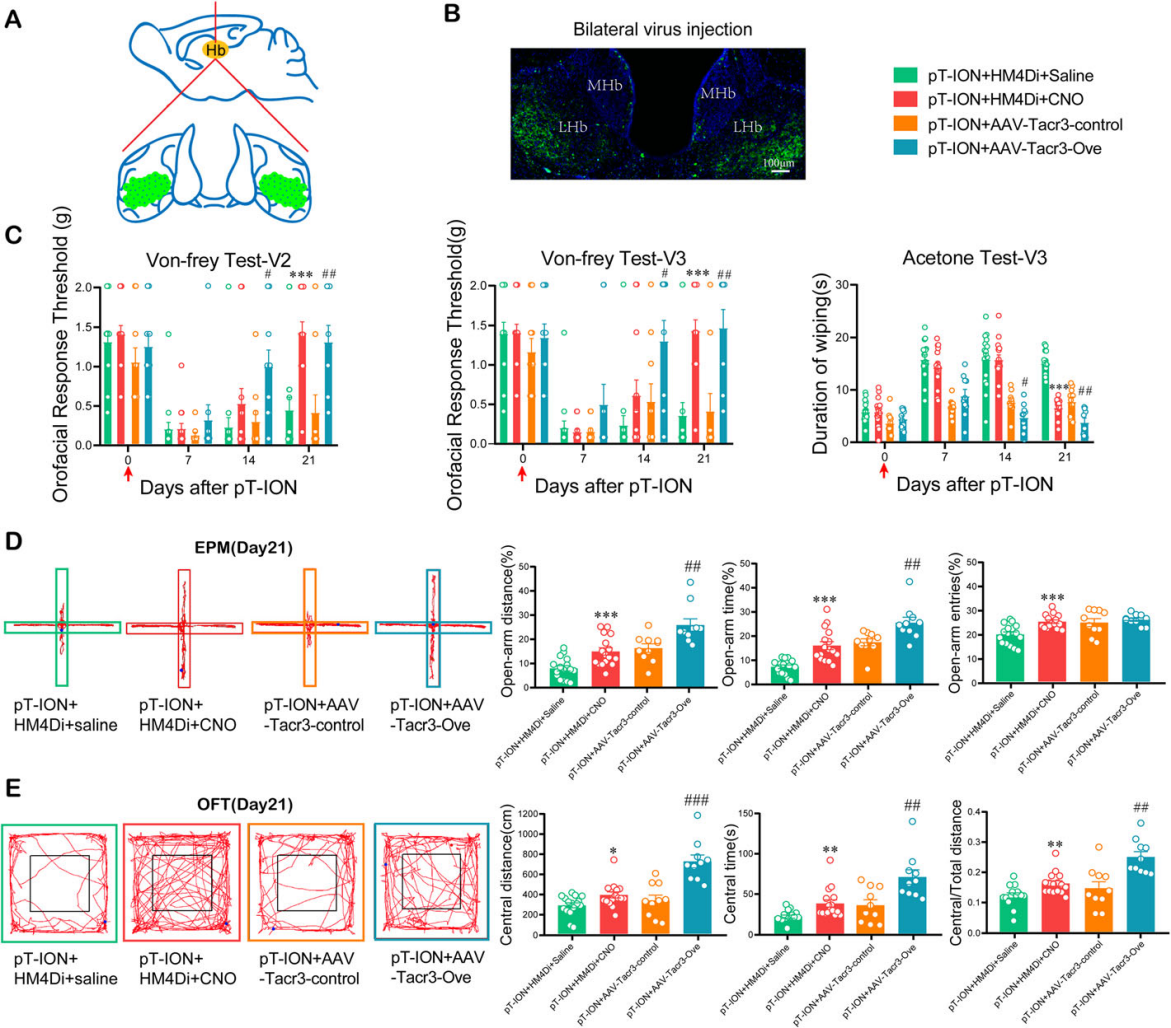
Inhibition of bilateral LHb by chemicogenetic intervention or *Tacr3* overexpression reversed pT-ION-induced allodynia and anxiety-like behaviors. **(a)** Schematic diagram showing the virus injection sites in the LHb. The green areas and black dots represent virus injection sites. **(b)** Typical immunofluorescence staining showing the virus transfection location in the bilateral LHb. Green, EGFP; Blue, DAPI. **(c)** The primary mechanical allodynia (left panel), secondary mechanical allodynia (middle panel), and secondary cold allodynia (right panel) were improved by bilateral HM4Di-CNO application and by *Tacr3* overexpression. *** *p* < 0.001 vs. the pT-ION+HM4Di + Saline group, # *p* < 0.05, ## *p* < 0.01 vs. the pT-ION+AAV-*Tacr3*-control group. **(d)** The decreases in percent of open-arm distance, time, and entries were completely reversed by bilateral HM4Di-CNO application and by *Tacr3* overexpression. *** *p* < 0.001 vs. the pT-ION+HM4Di + Saline group; ## *p* < 0.01 vs. the pT-ION+AAV-Tacr3-control group. **(e)** The central distance, central time, and central/total distance were significantly increased by bilateral HM4Di-CNO application and by *Tacr3* overexpression. *p < 0.05, ** *p* < 0.01 vs. the pT-ION+HM4Di + Saline group; ## *p* < 0.05, ### *p* < 0.01 vs. the pT-ION+AAV-*Tacr3*-control group

A previous study showed that unilateral nociceptive stimulation activated bilateral LHb [38]. We proposed that although the pT-ION-induced anxiety-like behaviors were significantly improved after inhibition of the unilateral LHb neurons, this might not be enough to reverse allodynia. Therefore we performed bilateral intervention of LHb neurons. First, we chemicogenetically inhibited the excitability of bilateral LHb neurons with HM4Di-CNO (Fig. 9a,b). Both the decrease in thresholds for mechanical stimulation in the V2/V3 skin areas and the increase in wiping duration after acetone application in the V3 skin area were significantly reversed (Fig. 9c). As we expected, compared with the pT-ION+HM4Di + Saline group, both the EPM and OFT suggested that inhibition of the hyperexcitation in bilateral LHb neurons significantly alleviated the nerve injury-induced anxiety-like behaviors (Fig. 9d,e). Second, we overexpressed *Tacr3* by injecting AAV-*Tacr3*-Ove virus into the bilateral LHb. Compared with the pT-ION+AAV-*Tacr3*-control group, the primary and secondary mechanical allodynia as well as the secondary cold allodynia were alleviated by the rescue of *Tacr3* downregulation by AAV-*Tacr3*-Ove virus (Fig. 9c). Both the EPM and OFT showed that the overexpression of *Tacr3* in bilateral LHb neurons could also reverse nerve injury-induced anxiety-like behaviors (Fig. 9d,e).

## Discussion

It has been shown that the LHb participates in the modulation of both pain and mood disorders and that removal of the LHb reduces both pain and mood disorders [23]. Here we provide the first demonstration that selective inhibition of glutamatergic neurons in the unilateral LHb alleviates pT-ION-induced anxiety-like behaviors but not allodynia, while both allodynia and anxiety-like behaviors are significantly alleviated by bilateral chemicogenetic inhibition of LHb or *Tacr3* overexpression, indicating that the LHb regulates allodynia and anxiety-like behaviors through different mechanisms. Furthermore, overexpression of *Tacr3* downregulated p-CaMKII, a marker of neuronal activation. In addition, whole-cell patch clamp recording showed that the nerve injury-induced increase in the amplitude and frequency of sEPSCs in LHb neurons was reversed by *Tacr3* overexpression, indicating the inhibition of LHb neurons by *Tacr3* overexpression. We propose that *Tacr3*-targeted interventions might be worth pursuing for treating pain and anxiety in TN.

In previous studies, the regulation of anxiety was more through bilateral intervention of habenula (Hb) function [8, 24, 35]. Here we observed the interesting phenomenon that unilateral inhibition of the excitability of the LHb was enough to alleviate pT-ION-induced anxiety-like behaviors, which means that less invasive interventions can be therapeutic. One of the possible reasons for this might be the prominent left-right asymmetry of the LHb. The left and right LHbs have some functions in common, but also have unique functions, and differences in the morphology, molecular organization, and even input/output circuits between the left and right Hb have been proposed [10, 14], although the left-right asymmetry of the Hb remains poorly understood. Stress is a common factor that can induce anxiety, and the Hb is asymmetrically activated in postnatal mice under stress [16]. Thus the left-right asymmetry might also explain the anti-anxiety effects of the unilateral LHb intervention observed in the present study. Clarifying the differences between the left and right circuits in the LHb will be key for a better understanding of their possible functions in different diseases, thus generating potential therapeutics for emotional disorders. Given that the human LHb is significantly large (95% of the total Hb volume) and that the left LHb is much larger than the right LHb in both genders [1, 13], unilateral intervention (i.e. the left side) might be considered for treating pain-associated anxiety and even pain in TN patients.

Studies on the contribution of the LHb to orofacial pain modulation are scarce, and the present study found that inhibition of glutamatergic neurons in the unilateral LHb did not reverse the pT-ION-induced allodynia. The LHb receives pain inputs from direct afferents via the trigeminal nucleus [11, 37] and sends its projections to the dorsal raphe nucleus [6, 41]. The dorsal raphe nucleus in turn sends afferents directly into the spinal trigeminal nucleus to contribute to descending serotoninergic inhibition [4]. Thus the inhibition of the glutamatergic neurons of the affected side in the LHb should have alleviated pT-ION-induced allodynia; however, our results showed that only anxiety-like behaviors were significantly reversed and not allodynia. A previous study showed that unilateral nociceptive stimulation induced the bilateral activation of the LHb [38], and thus the unilateral inhibition of glutamatergic neurons in the LHb by HM4Di-CNO intervention might not have sufficiently eliminated the effects of LHb modulation in the development of pT-ION-induced allodynia. Thus we inhibited bilateral LHb glutamatergic neurons and found that pT-ION-induced allodynia was significantly alleviated. In addition, the lack of analgesic effects after glutamatergic neuron inhibition by HM4Di-CNO intervention in the present study might also be due to different levels of inhibition of the subnuclear regions of the unilateral LHb. The LHb is divided into several subnuclei marked with various neurochemical contents [42], and this anatomic heterogeneity is also seen in humans [40]. Therefore, further understanding of the connectivity and function of the LHb on each side and the included subnuclei would be helpful for elucidating the role of the LHb in pain modulation.

Gene expression changes have been shown to provide potential targets for developing therapeutic agents for pain and anxiety in humans [15, 32]. In the present study, we primarily observed the function and gene expression adaptation of the left LHb (ipsilateral to the pT-ION). We found that *Tacr3* in the left LHb was among the top 20 most strongly down-regulated DEGs, and the downregulation of *Tacr3* was specifically seen in the LHb and not in other negative emotion-related brain areas such as the ACC, PFC, and hippocampus. Moreover, *Tacr3* overexpression could reverse the trigeminal nerve injury-induced allodynia and anxiety-like behaviors by inhibiting the hyperexcitability of LHb neurons. *Tacr3* belongs to the gene family that encodes receptors for tachykinins [28], and *Tacr3*^−/−^ mice were reported to have central reproductive defects [44]. *Tacr3*-encoded NK3R interacts with G proteins and is primarily expressed in the central nervous system [31]. Although relatively little is known about the behavioral and pharmacological mechanisms mediated by NK3R, recent results suggest an involvement in processes underlying learning and memory as well as emotionality [39, 47], and previous studies showed that the activation of NK3R might be necessary for the maintenance of pain hypersensitivity [18]. In addition, NK3R intervention showed good therapeutic effects on improving negative emotion such as depression; however, the results were unexpectedly contradictory. The NK3R agonist aminosenktide was reported to show antidepressant activity in mice [33], while the NK3R antagonist osanetant was also shown to have antidepressant activity in gerbils [36]. This unexpected similarity in effect from the two types of compounds might in part be due to differences between species. Neurokinin B (NKB), the endogenous ligand for NK3R, very possibly comes from the presynaptic terminals projecting from the medial habenula (MHb), and asymmetrical MHb projections to the LHb have been confirmed [19]. Previous studies have shown numerous NKB-positive cells in the MHb [29, 30], but for the LHb only the fibers and terminals are NKB positive and no NKB-immunoreactivity perikarya or mRNA are seen [30]. From these studies, it is reasonable to assume that NKB is released from the terminals coming from the MHb and that this inhibits the pT-ION-induced activation of LHb neurons by combining with postsynaptic NK3R. This process ultimately reverses allodynia and anxiety-like behaviors after nerve injury.

Previous animal research has been shown to be translatable to humans. For example, functional connectivity between the periaqueductal gray (the key structure involved in descending pain modulation) and the LHb has been identified in humans, and the bilateral activation of the LHb upon nociceptive stimulation has also been demonstrated in humans [38]. The LHb characteristics in terms of both structure and function are translatable from rodents to humans, and gene expression in the LHb in humans and rodents serves as another piece of evidence for the translational potential for LHb research [7]. It is worthwhile to undertake further experimental investigations to verify the role of *Tacr3* in the LHb in allodynia and anxiety-like behaviors in TN. Hopefully more human research will be performed in the future in order to support the translation from animal research to clinical practice.

## Conclusions

In conclusion, our results indicate that trigeminal nerve injury induces stable and long-lasting orofacial allodynia and anxiety-like behaviors and is correlated with hyperexcitation of LHb neurons and gene expression adaptation. *Tacr3*, a gene belonging to the calcium signaling pathway, was downregulated after nerve injury. Functional inhibition of the unilateral LHb by chemicogenetic methods or by rescuing the downregulation of *Tacr3* could alleviate nerve injury-induced anxiety-like behaviors but not allodynia, and significant reversal of both allodynia and anxiety-like behaviors required the bilateral inhibition of the LHb. Therefore, nerve injury-induced allodynia and anxiety-like behaviors are differentially modulated by the LHb, and selective inhibition of unilateral or bilateral LHb function by *Tacr3*-targeting methods is a promising treatment for pain and anxiety in TN patients.

## Supplementary information

**Additional file 1: Figure S1.** Chemicogenetic inhibition of the left LHb did not change basal pain threshold or parameters for anxiety-like behaviors in sham mice. **(a)** Thresholds for mechanical stimulation in the V2 (left) and V3 (middle) area and the wiping time in response to acetone (right) were unchanged by HM4Di injection in the left LHb and the subsequent CNO application. **(b)** The percentages of open-arm distance, time, and entries were unchanged following the inhibition of left LHb neurons by HM4Di-CNO. **(c)** The central distance, central time, and central/total distance in the OFT were unchanged following the inhibition of left LHb neurons by HM4Di-CNO.

**Additional file 2: Figure S2.** The down-regulation of *Tacr3* expression is specifically located in the LHb. The expression of *Tacr3* was unchanged in the ACC **(a)**, PFC **(b),** and hippocampus **(c)** after pT-ION.

**Additional file 3: Figure S3.***Tacr3* overexpression in the left LHb did not change the basal pain threshold or parameters for anxiety-like behaviors in sham mice. **(a)** Thresholds for mechanical stimulation in the V2 (left) and V3 (middle) areas and wiping time in response to acetone (right) were unchanged by AAV-*Tacr3*-ove application. **(b)** The percentages of open-arm distance, time, and entries were unchanged following AAV-*Tacr3*-ove application in the left LHb. **(c)** The central distance, central time, and central/total distance in the OFT were unchanged by AAV-*Tacr3*-ove application in the left LHb.

**Additional file 4: Table S1.** Statistical analysis results for two-way RM ANOVA. **Table S2** Statistical analysis results for one-way ANOVA. **Table S3** Statistical analysis results for Student’s t-test. **Table S4** The top 20 DEGs in the LHb.

## Data Availability

The datasets used in the current study are available from the corresponding author on reasonable request.

## Abbreviations

TN: Trigeminal neuralgia
LHb: Lateral habenula
pT-ION: Partial transection of the infraorbital nerve
DEGs: Differentially expressed genes
CNO: Clozapine-N-oxide
GO: Gene Ontology
KEGG: Kyoto Encyclopedia of Genes and Genomes
CCI-ION: Chronic constriction injury of the infraorbital nerve
OFT: Open field test
EPM: Elevated plus maze
NK3R: Neurokinin 3 receptor
NKB: Neurokinin B
MHb: Medial habenula
