# Supplementary figures and images for: Tacr3 in the lateral habenula differentially regulates orofacial allodynia and anxiety-like behaviors in a mouse model of trigeminal neuralgia
Source: Acta Neuropathol Commun. 2020 Apr 7;8:44. doi: 10.1186/s40478-020-00922-9 (PMC7137530; doi:10.1186/s40478-020-00922-9)

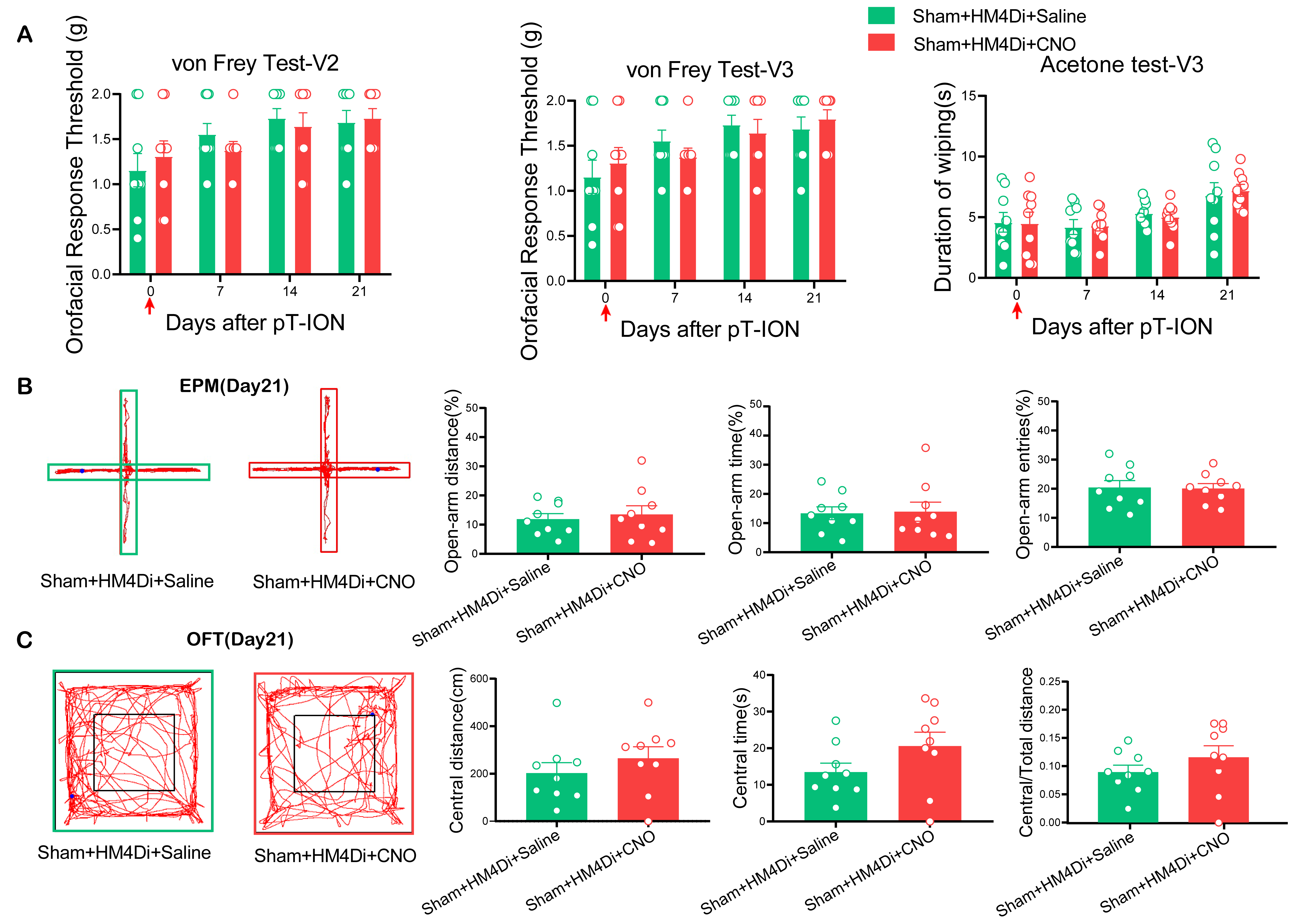

Supplement: Supplementary file 1 — Additional file 1: Figure S1. Chemicogenetic inhibition of the left LHb did not change basal pain threshold or parameters for anxiety-like behaviors in sham mice. (a) Thresholds for mechanical stimulation in the V2 (left) and V3 (middle) area and the wiping time in response to acetone (right) were unchanged by HM4Di injection in the left LHb and the subsequent CNO application. (b) The percentages of open-arm distance, time, and entries were unchanged following the inhibition of left LHb neurons by HM4Di-CNO. (c) The central distance, central time, and central/total distance in the OFT were unchanged following the inhibition of left LHb neurons by HM4Di-CNO. [file 40478_2020_922_MOESM1_ESM.tif]

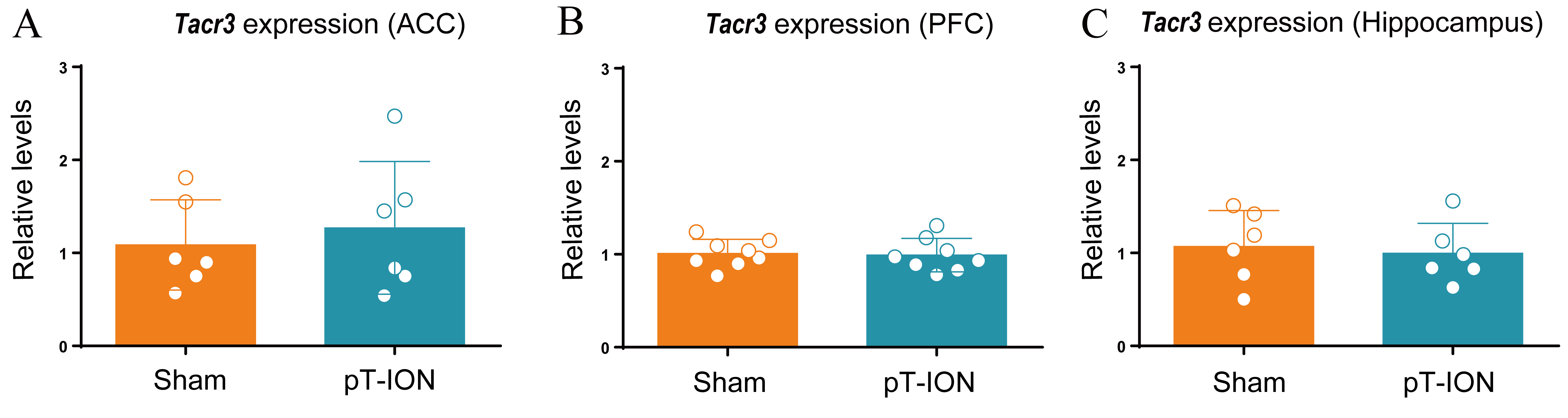

Supplement: Supplementary file 2 — Additional file 2: Figure S2. The down-regulation of Tacr3 expression is specifically located in the LHb. The expression of Tacr3 was unchanged in the ACC (a), PFC (b), and hippocampus (c) after pT-ION. [file 40478_2020_922_MOESM2_ESM.tif]

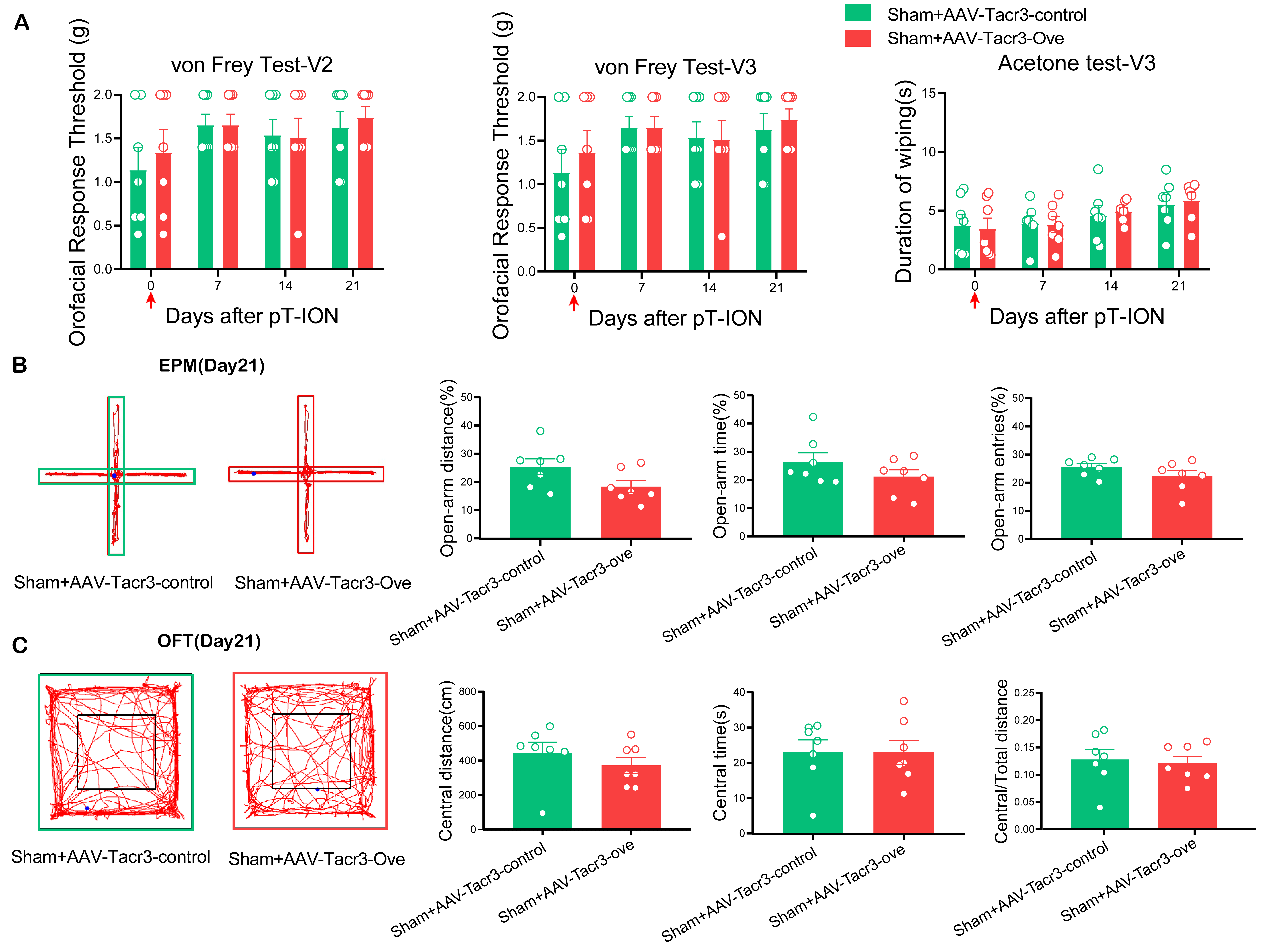

Supplement: Supplementary file 3 — Additional file 3: Figure S3.Tacr3 overexpression in the left LHb did not change the basal pain threshold or parameters for anxiety-like behaviors in sham mice. (a) Thresholds for mechanical stimulation in the V2 (left) and V3 (middle) areas and wiping time in response to acetone (right) were unchanged by AAV-Tacr3-ove application. (b) The percentages of open-arm distance, time, and entries were unchanged following AAV-Tacr3-ove application in the left LHb. (c) The central distance, central time, and central/total distance in the OFT were unchanged by AAV-Tacr3-ove application in the left LHb. [file 40478_2020_922_MOESM3_ESM.tif]
