# Supplementary material for: Tacr3 in the lateral habenula differentially regulates orofacial allodynia and anxiety-like behaviors in a mouse model of trigeminal neuralgia
Source: Acta Neuropathol Commun. 2020 Apr 7;8:44. doi: 10.1186/s40478-020-00922-9 (PMC7137530; doi:10.1186/s40478-020-00922-9)
Supplement: Supplementary file 4 — Additional file 4: Table S1. Statistical analysis results for two-way RM ANOVA. Table S2 Statistical analysis results for one-way ANOVA. Table S3 Statistical analysis results for Student’s t-test. Table S4 The top 20 DEGs in the LHb. [file 40478_2020_922_MOESM4_ESM.docx]

Supplementary Information for

***Tacr3* in the lateral habenula differentially regulates orofacial allodynia and anxiety-like behaviors in a mouse model of trigeminal neuralgia**

Figure and table legends

**Fig. S1 Chemicogenetic inhibition of the left LHb did not change basal pain threshold or parameters for anxiety-like behaviors in sham mice. (a)** Thresholds for mechanical stimulation in the V2 (left) and V3 (middle) area and the wiping time in response to acetone (right) were unchanged by HM4Di injection in the left LHb and the subsequent CNO application. **(b)** The percentages of open-arm distance, time, and entries were unchanged following the inhibition of left LHb neurons by HM4Di-CNO. **(c)** The central distance, central time, and central/total distance in the OFT were unchanged following the inhibition of left LHb neurons by HM4Di-CNO.

**Fig. S2** **The down-regulation of *Tacr3* expression is specifically located in the LHb.** The expression of *Tacr3* was unchanged in the ACC **(a)**, PFC **(b),** and hippocampus **(c)** after pT-ION.

**Fig. S3 *Tacr3* overexpression in the left LHb did not change the basal pain threshold or parameters for anxiety-like behaviors in sham mice. (a)** Thresholds for mechanical stimulation in the V2 (left) and V3 (middle) areas and wiping time in response to acetone (right) were unchanged by AAV-*Tacr3*-ove application. **(b)** The percentages of open-arm distance, time, and entries were unchanged following AAV-*Tacr3*-ove application in the left LHb. **(c)** The central distance, central time, and central/total distance in the OFT were unchanged by AAV-*Tacr3*-ove application in the left LHb.

**Table S1 Statistical analysis results for two-way RM ANOVA**

| Figure | Panel | Interaction  DFn,  DFd F *p* | | | Time  DFn,  DFd F *p* | | | Treatment  DFn,  DFd F *p* | | |
| --- | --- | --- | --- | --- | --- | --- | --- | --- | --- | --- |
| 1 | A | 6,63 | 31.83 | <0.0001 | 3,63 | 43.20 | <0.0001 | 2,21 | 331.00 | <0.0001 |
| 1 | B | 6,63 | 61.82 | <0.0001 | 3,63 | 59.06 | <0.0001 | 2,21 | 334.20 | <0.0001 |
| 1 | C | 6,63 | 69.06 | <0.0001 | 3,63 | 21.59 | <0.0001 | 2,21 | 39.22 | <0.0001 |
| 5 | D_1_ | 9,246 | 87.50 | <0.0001 | 3,246 | 774.30 | <0.0001 | 3,82 | 151.90 | <0.0001 |
| 5 | D_2_ | 9,246 | 70.85 | <0.0001 | 3,246 | 690.90 | <0.0001 | 3,82 | 145.60 | <0.0001 |
| 5 | D_3_ | 9,246 | 50.72 | <0.0001 | 3,246 | 488.10 | <0.0001 | 3,82 | 40.42 | <0.0001 |
| 7 | D_1_ | 9,228 | 74.92 | <0.0001 | 3,228 | 636.60 | <0.0001 | 3,76 | 138.00 | <0.0001 |
| 7 | D_2_ | 9,228 | 75.11 | <0.0001 | 3,228 | 701.60 | <0.0001 | 3,76 | 135.80 | <0.0001 |
| 7 | D_3_ | 9,228 | 26.93 | <0.0001 | 3,228 | 247.40 | <0.0001 | 3,76 | 44.04 | <0.0001 |
| 9 | C_1_.HM4Di | 390 | 6.826 | 0.0003 | 390 | 38.97 | <0.0001 | 130 | 9.705 | 0.0040 |
| 9 | C_1_.*Tacr3* | 354 | 2.205 | 0.0980 | 354 | 10.74 | <0.0001 | 118 | 12.73 | 0.0022 |
| 9 | C_2_.HM4Di | 390 | 8.961 | <0.0001 | 390 | 39.35 | <0.0001 | 130 | 10.52 | 0.0029 |
| 9 | C_2_.*Tacr3* | 354 | 1.859 | 0.1476 | 354 | 7.097 | 0.0004 | 118 | 7.097 | 0.0008 |
| 9 | C_3_.HM4Di | 390 | 13.20 | <0.0001 | 390 | 68.14 | <0.0001 | 130 | 17.26 | 0.0002 |
| 9 | C_3_.*Tacr3* | 354 | 6.730 | 0.0004 | 354 | 6.849 | 0.0005 | 118 | 2.984 | 0.1012 |
| S1 | A_1_ | 348 | 0.5508 | 0.6500 | 348 | 5.117 | 0.0037 | 116 | 0.03636 | 0.8512 |
| S1 | A_2_ | 348 | 0.6793 | 0.5690 | 348 | 5.832 | 0.0018 | 116 | 0 | >0.9999 |
| S1 | A_3_ | 348 | 0.09064 | 0.9648 | 348 | 6.006 | 0.0015 | 116 | 0.01090 | 0.9181 |
| S3 | A_1_ | 336 | 0.1859 | 0.9053 | 336 | 2.729 | 0.0582 | 112 | 0.1912 | 0.6697 |
| S3 | A_2_ | 336 | 0.2398 | 0.8680 | 336 | 2.659 | 0.0629 | 112 | 0.2333 | 0.6378 |
| S3 | A_3_ | 336 | 0.1263 | 0.9439 | 336 | 4.465 | 0.0091 | 112 | 0.007919 | 0.9306 |

**Table S2 Statistical analysis results for one-way ANOVA**

| Figure Panel Factor | | | Treatment  DFn,  DFd F *p* | | | Treatment  Figure Panel Factor DFn,  DFd F *p* | | | | | | |
| --- | --- | --- | --- | --- | --- | --- | --- | --- | --- | --- | --- | --- |
| 2 | A_1_ | Open-arm distance | 2,21 | 1.12 | =0.3453 |  | 3 | C_1_ | Central distance | 2,21 | 60.97 | <0.0001 |
| 2 | A_2_ | Open-arm time | 2,21 | 1.10 | =0.3503 |  | 3 | C_2_ | Central time | 2,21 | 58.62 | <0.0001 |
| 2 | A_3_ | Open-arm entries | 2,21 | 1.14 | =0.3390 |  | 3 | C_3_ | Central/total distance | 2,21 | 3.64 | =0.0440 |
| 2 | B_1_ | Open-arm distance | 2,21 | 15.45 | <0.0001 |  | 5 | E_1_ | Central distance | 3,82 | 8.21 | <0.0001 |
| 2 | B_2_ | Open-arm time | 2,21 | 14.18 | =0.0001 |  | 5 | E_2_ | Central time | 3,82 | 4.76 | =0.0041 |
| 2 | B_3_ | Open-arm entries | 2,21 | 10.16 | =0.0008 |  | 5 | E_3_ | Central/total distance | 3,82 | 6.09 | =0.0009 |
| 2 | C_1_ | Open-arm distance | 2,21 | 20.21 | <0.0001 |  | 5 | F_1_ | Central distance | 3,82 | 11.36 | <0.0001 |
| 2 | C_2_ | Open-arm time | 2,21 | 34.13 | <0.0001 |  | 5 | F_2_ | Central time | 3,82 | 14.37 | <0.0001 |
| 2 | C_3_ | Open-arm entries | 2,21 | 51.59 | <0.0001 |  | 5 | F_3_ | Central/total distance | 3,82 | 6.02 | =0.0009 |
| 3 | A_1_ | Central distance | 2,21 | 0.66 | =0.5250 |  | 7 | C | Tacr3 | 3,12 | 11.42 | =0.0008 |
| 3 | A_2_ | Central time | 2,21 | 0.47 | =0.6313 |  | 7 | E_1_ | Open-arm distance | 3,76 | 9.43 | <0.0001 |
| 3 | A_3_ | Central/total distance | 2,21 | 0.33 | =0.7215 |  | 7 | E_2_ | Open-arm time | 3,76 | 9.66 | <0.0001 |
| 3 | B_1_ | Central distance | 2,21 | 23.43 | <0.0001 |  | 7 | E_3_ | Open-arm entries | 3,76 | 10.57 | <0.0001 |
| 3 | B_2_ | Central time | 2,21 | 14.83 | <0.0001 |  | 7 | F_1_ | Central distance | 3,76 | 11.95 | <0.0001 |
| 3 | B_3_ | Central/total distance | 2,21 | 5.61 | =0.0112 |  | 7 | F_2_ | Central time | 3,76 | 7.58 | =0.0002 |
|  |  |  |  |  |  |  | 7 | F | Central/total distance | 3,76 | 5.73 | =0.0014 |

**Table S3 Statistical analysis results for Student’s t-test**

| Figure | Panel | Comparison | Student’s T-test T *p* | | | | DFn | DFd |
| --- | --- | --- | --- | --- | --- | --- | --- | --- |
| 4 | A | p-CaMKⅡ | | Unpaired | 3.50 | 0.0129 | 3 | 3 |
| 4 | C | Amplitude of sEPSC | | Unpaired | 3.001 | 0.0075 | 9 | 8 |
| 4 | D | Frequency of sEPSC | | Unpaired | 4.901 | <0.0001 | 10 | 9 |
| 6 | G | *Tacr3* expression | | Unpaired | 5.64 | 0.0013 | 3 | 3 |
| 8 | A | Ove vs. Control | | Unpaired | 3.28 | 0.0168 | 3 | 3 |
| 8 | A | CNO vs. Saline | | Unpaired | 4.02 | 0.0069 | 3 | 3 |
| 8 | C | Amplitude of sEPSC | | Unpaired | 2.333 | 0.0302 | 9 | 11 |
| 8 | D | Frequency of sEPSC | | Unpaired | 3.558 | 0.0020 | 9 | 11 |
| 9 | D_1_ | CNO vs. Saline | | Unpaired | 3.693 | 0.0009 | 9 | 9 |
| 9 | D_1_ | Ove vs. Control | | Unpaired | 3.096 | 0.0062 | 15 | 15 |
| 9 | D_2_ | CNO vs. Saline | | Unpaired | 4.811 | <0.0001 | 9 | 9 |
| 9 | D_2_ | Ove vs. Control | | Unpaired | 3.063 | 0.0067 | 15 | 15 |
| 9 | D_3_ | CNO vs. Saline | | Unpaired | 4.276 | 0.0002 | 9 | 9 |
| 9 | D_3_ | Ove vs. Control | | Unpaired | 0.6967 | 0.4941 | 15 | 15 |
| 9 | E_1_ | CNO vs. Saline | | Unpaired | 2.663 | 0.0123 | 9 | 9 |
| 9 | E_1_ | Ove vs. Control | | Unpaired | 4.691 | 0.0002 | 15 | 15 |
| 9 | E_2_ | CNO vs. Saline | | Unpaired | 3.290 | 0.0026 | 9 | 9 |
| 9 | E_2_ | Ove vs. Control | | Unpaired | 3.117 | 0.0060 | 15 | 15 |
| 9 | E_3_ | CNO vs. Saline | | Unpaired | 3.219 | 0.0031 | 9 | 9 |
| 9 | E_3_ | Ove vs. Control | | Unpaired | 3.694 | 0.0017 | 15 | 15 |
| S1 | B_1_ | Open-arm distance | | Unpaired | 0.4518 | 0.6575 | 8 | 8 |
| S1 | B_2_ | Open-arm time | | Unpaired | 0.1391 | 0.8911 | 8 | 8 |
| S1 | B_3_ | Open-arm entries | | Unpaired | 0.1168 | 0.9085 | 8 | 8 |
| S1 | C_1_ | Central distance | | Unpaired | 0.9564 | 0.3531 | 8 | 8 |
| S1 | C_2_ | Central time | | Unpaired | 1.573 | 0.1354 | 8 | 8 |
| S1 | C_3_ | Central/total distance | | Unpaired | 1.101 | 0.2872 | 8 | 8 |
| S2 | A | ACC | | Unpaired | 0.5203 | 0.6142 | 5 | 5 |
| S2 | B | PFC | | Unpaired | 0.2267 | 0.8239 | 7 | 7 |
| S2 | C | Hippocampus | | Unpaired | 0.3646 | 0.7230 | 5 | 5 |
| S3 | B_1_ | Open-arm distance | | Unpaired | 1.992 | 0.0697 | 6 | 6 |
| S3 | B_2_ | Open-arm time | | Unpaired | 1.322 | 0.2109 | 6 | 6 |
| S3 | B_3_ | Open-arm entries | | Unpaired | 1.429 | 0.1785 | 6 | 6 |
| S3 | C_1_ | Central distance | | Unpaired | 0.9533 | 0.3592 | 6 | 6 |
| S3 | C_2_ | Central time | | Unpaired | 001479 | 0.9884 | 6 | 6 |
| S3 | C_3_ | Central/total distance | | Unpaired | 0.3118 | 0.7605 | 6 | 6 |

**Table S4 The top 20 DEGs in the LHb**

| ID | Ensembl ID | Entrez Gene | Gene Symbol | Gene Name | FC | P.  Value | Stage |
| --- | --- | --- | --- | --- | --- | --- | --- |
| NM-008552 | NA | 17171 | Mas 1 | MAS1 oncogene | 12.37 | 0.0188 | up |
| NM-001081078 | ENSMUST00000073490 | 226413 | Lct | Lactase | 11.01 | 0.0184 | up |
| NM-001160112 | ENSMUST00000021333 | 15228 | Foxg 1 | Forkhead box G1 | 7.34 | 0.0227 | up |
| NM-018827 | ENSMUST00000132648 | 12931 | Crlf 1 | Cytokine receptor-like factor 1 | 6.37 | 0.0381 | up |
| NM-029947 | ENSMUST00000112959 | 77630 | Prdm 8 | PR domain containing 8 | 6.08 | 0.0347 | up |
| NM-080433 | ENSMUST00000022262 | 54713 | Fezf 2 | Fez family zinc finger 2 | 6.01 | 0.0389 | up |
| NM-023842 | ENSMUST00000124830 | 109620 | Dsp | Desmoplakin | 5.84 | 0.0401 | up |
| NM-012045 | ENSMUST00000030526 | 26971 | Pla2g2f | “phospholipase A2, group IIF” | 5.73 | 0.0134 | up |
| NM-139218 | ENSMUST00000049644 | 73708 | Dppa 3 | Developmental pluripotency-associated 3 | 5.62 | 0.0411 | up |
| NM-201255 | ENSMUST00000059707 | 107656 | Krt 9 | Keratin 9 | 5.55 | 0.0135 | up |
| NM-177187 | ENSMUST00000094593 | 320549 | D5Ertd577e | “DNA segment, Chr 5, ERATO Doi 577, expressed | 5.38 | 0.0156 | up |
| NA | ENSMUST00000034133 | 213435 | Mylk 3 | Myosin light chain kinase 3 | 5.15 | 0.0333 | up |
| NM-016669 | ENSMUST00000033198 | 12971 | Crym | “crystalline, mu” | 4.93 | 0.0207 | up |
| XR-403899 | NA | 675749 | Gm10693 | Predicted pseudogene 10693 | 4.83 | 0.0318 | up |
| NM-146962 | ENSMUST00000080681 | 258964 | Olfr541 | Olfactory receptor 541 | 4.23 | 0.0298 | up |
| NM-023456 | ENSMUST00000031843 | 109648 | Npy | Neuropeptide Y | 3.87 | 0.0237 | up |
| NA | NA | 101055709 | Zfp264 | Zinc finger protein 264 | 3.85 | 0.0461 | up |
| NM-146125 | ENSMUST00000028758 | 228550 | Itpka | “inositol 1,4,5-trisphosphate 3-kinase A” | 3.83 | 0.0157 | up |
| NM-013838 | NA | 22068 | Trpc 6 | “transient receptor potential cation channel, subfamily C, member 6” | 3.77 | 0.0402 | up |
| NM-011088 | ENSMUST00000108615 | 18724 | Pira 11 | Paired-lg-like receptor A11 | 3.72 | 0.0198 | up |
| NM-001177964 | NA | 68511 | Dcdc2c | Doublecortin domain containing 2C | -2.37 | 0.0167 | down |
| NM-177372 | ENSMUST00000129785 | 327762 | Dna 2 | DNA replication helicase 2 homolog | -2.39 | 0.0099 | down |
| NM-029906 | ENSMUST00000020392 | 77397 | 9530003J23Rik | RIKEN cDNA 9530003J23 gene | -2.43 | 0.0270 | down |
| NM-139220 | ENSMUST00000047851 | 246080 | Defb 7 | Defensin receptor 3 | -2.45 | 0.0152 | down |
| NM-023872 | ENSMUST00000174183 | 226922 | Kcnq 5 | “potassium voltage-gated channel, subfamily Q, member 5” | -2.46 | 0.0013 | down |
| NM-021382 | ENSMUST00000029822 | 21338 | Tacr 3 | Tachykinin receptor 3 | -2.46 | 0.0242 | down |
| NM-001081129 | ENSMUST00000091554 | 238680 | Cntnap 3 | Contactin associated protein-like 3 | -2.51 | 0.0222 | down |
| NM-001001179 | ENSMUST00000060574 | 232400 | BC048546 | cDNA sequence BC048546 | -2.54 | 0.0188 | down |
| NM-029290 | NA | 75444 | 1700011I03Rik | RIKEN cDNA 1700011I03 gene | -2.56 | 0.0311 | down |
| NM-008267 | ENSMUST00000062709 | 15408 | Hoxb 13 | Homeobox B13 | -2.59 | 0.0347 | down |
| NM-175280 | ENSMUST00000133433 | 78774 | 4930529M08Rik | RIKEN cDNA 4930529M08 | -2.64 | 0.0155 | down |
| NM-203492 | ENSMUST00000058092 | 381974 | Mrgprg | “MAS-related GPR, member G” | -2.67 | 0.0074 | down |
| NM-026253 | ENSMUST00000038956 | 67580 | Lrrc 18 | Leucine rich repeat containing 18 | -2.71 | 0.0158 | down |
| NM-001025240 | ENSMUST00000056023 | 384813 | Gm5346 | Predicted gene 5346 | -2.78 | 0.0303 | down |
| XR-401822 | NA | 78687 | 0610025J13Rik | RIKEN cDNA 0610025J13 gene | -3.03 | 0.0349 | down |
| NM-013756 | NA | 27358 | Defb 3 | Defensin beta 3 | -3.10 | 0.0176 | down |
| NM-130869 | ENSMUST00000031749 | 18291 | Nobox | NOBOX oogenesis homeobox | -3.64 | 0.0380 | down |
| NM-011475 | ENSMUST00000047264 | 20763 | Sprr2i | Small proline-rich protein 2I | -3.90 | 0.0018 | down |
| NA | ENSMUST00000051236 | 320982 | Arl4c | ADP-ribosylation factor-like 4C | -4.15 | 0.0002 | down |
| **NM-011472** | **ENSMUST00000050397** | **20760** | **Sprr2f** | **Small proline-rich protein 2F** | **-5.55** | **0.0001** | **down** |
